# MLFRNet: Multi-Scale Local Feature Refinement Network Using Multi-Scale Depthwise Convolution for HER2 Score Classification

**DOI:** 10.3390/diagnostics16213614

**Published:** 2026-11-01

**Authors:** Mohan Venkata Sai Valluru, Bala Venkateswarlu Isunuri, Sibendu Samanta

**Affiliations:** 1 Department of Computer Science and Engineering, School of Engineering and Sciences, SRM University-AP, Amaravati 522240, Andhra Pradesh, India; mohanvenkatasai_v@srmap.edu.in; 2 Department of Electronics and Communication Engineering, School of Engineering and Sciences, SRM University-AP, Amaravati 522240, Andhra Pradesh, India; sibendu.s@srmap.edu.in

**Keywords:** multi-scalelocal feature refinement network, multi-scale depthwise convolution, HER2 score classification, histopathology image classification

## Abstract

**Background/Objectives**: Human Epidermal Growth Factor Receptor 2 (HER2) is a key biomarker for selecting treatment strategies in breast cancer patients. In routine pathology workflows, HER2 assessment is performed by pathologists through examination of immunohistochemistry (IHC)-stained sections under a microscope. Manual IHC assessment requires expert interpretation and can be subject to inter-observer variability, which motivates the study of computer-assisted analysis in digital pathology. **Methods**: MLFRNet uses a ConvNeXt-Tiny backbone with a Multi-scale Local Feature Refinement (MLFR) module that integrates Efficient Channel Attention (ECA), a multi-scale depthwise convolution block (MSB), and a residual refinement block (RB). ECA reweights channel representations without dimensionality reduction, after which MSB applies parallel depthwise convolutions with 3 × 3, 5 × 5, and 7 × 7 kernels to capture complementary spatial contexts. The resulting representation is further processed by RB before classification. Comprehensive experiments were conducted on the HER2-IHC-40× dataset across three prespecified stochastic runs. **Results**: The mean test accuracy was 96.10 ± 0.22% and the macro-averaged F1-score was 95.30 ± 0.23%. **Conclusions**: Because the public release does not provide patient or whole-slide identifiers, patient-level and slide-level independence of the partitions could not be independently verified. The reported results should therefore be interpreted as an exploratory patch-level benchmark rather than as patient-level or clinically validated HER2 scoring performance.

## 1. Introduction

Breast cancer remains the most prevalent malignancy affecting women worldwide [1]. According to global health statistics, breast cancer accounts for a significant proportion of cancer incidence among women [2,3]. For effective treatment and better survival rates for patients, it is important to identify and describe tumors early on. Among the biomarkers used for breast cancer diagnosis and treatment planning, Human Epidermal Growth Factor Receptor 2 (HER2) plays a crucial role [4,5]. HER2 overexpression promotes uncontrolled cellular proliferation, and accurate assessment of HER2 expression is relevant to treatment planning. HER2 expression is commonly evaluated by immunohistochemistry (IHC) staining of breast tissue obtained by biopsy, followed by assessment of membrane staining and related morphological features by expert pathologists. Under current diagnostic standards established by the American Society of Clinical Oncology (ASCO) and the College of American Pathologists (CAP) [6], IHC staining is assigned to four score categories: Score 0 (Negative), Score 1+ (Negative), Score 2+ (Equivocal), and Score 3+ (Positive). These IHC scores should be distinguished from final HER2 status, which may require additional clinical or molecular assessment, particularly for equivocal cases.

In routine clinical pathology laboratories, HER2 expression is assessed by IHC, which requires experienced pathologist interpretation. Recent advances in digital pathology and computer-aided diagnosis (CAD) have enabled CNN-based approaches for image analysis and may reduce the burden of repetitive visual assessment [7,8,9,10,11,12]. CNNs can automatically learn discriminative image features from histopathology data. Pretrained models, including VGGNet [8], ResNet [9], DenseNet [10], and EfficientNet [12], have been widely adopted using transfer learning techniques to improve classification performance in histopathological datasets. However, despite all these advances, histopathology image analysis remains challenging due to its complex tissue structure, staining intensity, and cellular arrangement. Histopathology images contain visual information at multiple spatial scales, ranging from cellular structures to larger tissue patterns.

Standard convolutional layers with a fixed kernel size may be limited in capturing hierarchical spatial information in complex histopathology images. This limitation has motivated the use of alternative approaches, such as dilated convolutions and attention mechanisms, that can capture features over multiple spatial extents. Consequently, multi-scale feature extraction has become an important research direction in histopathology image analysis. Multi-scale representations allow neural networks to learn from both local and broader contextual information. Architectures such as dilated-convolution frameworks and pyramid pooling can analyze images at different spatial scales, although they may increase computational complexity and resource requirements. More recently, ConvNeXt [11] has demonstrated strong performance in computer vision while retaining the spatial inductive bias of convolutional networks. Despite its strong feature-extraction capability, ConvNeXt does not explicitly model multi-scale spatial contexts. Motivated by this limitation, this study proposes a novel Multi-scale Local Feature Refinement Network (MLFRNet) for automated HER2 score classification. The proposed architecture builds upon the ConvNeXt-Tiny backbone and introduces multi-scale depthwise convolutions with different kernel sizes to capture complementary local spatial features across different neighborhood extents.

The remainder of this paper is organized as follows. Section 2 reviews related studies on histopathology image classification and HER2 assessment. Section 3 presents the proposed MLFRNet architecture and the experimental methodology, including the preprocessing, training configuration, loss function, evaluation metrics, and implementation details. Section 4 presents the dataset details and experimental results, including performance evaluation, ablation studies, visualization analyses, and computational characterization. Section 5 discusses the findings, their implications, and the limitations of the study. Finally, Section 6 concludes the paper and outlines directions for future research.

## 2. Related Work

Several approaches have been proposed for breast histopathology image classification. Early work mainly considered binary classification, whereas later studies addressed multiclass settings in which interclass similarity and intraclass variability increase the difficulty of discrimination. Nabi et al. [13] proposed a deep learning framework combining CNN and DenseNet121 with Gradient-weighted Class Activation Mapping(Grad-CAM) and SHapley Additive exPlanations (SHAP ) for interpretability. Pretrained architectures such as EfficientNet, DenseNet, and ResNet have also been widely used as feature-extraction backbones [9,10,12]. Because these architectures were developed primarily for natural images, additional attention and multi-scale mechanisms have been explored to better represent the heterogeneous spatial structure of histopathology images.

Recent advancements have shown that hybrid and transformer-based architectures improve multi-class classification [14]. An analysis by Sriwastawa et al. [15] demonstrated that attention-based models can effectively capture the global context information in histopathology images. Models such as Cross-View Vision Transformer (CrossViT), Maximum Vision Transformer (MaxViT), and Convolutional Vision Transformer (CvT) showed competitive performance over Breast Histopathology Images (BreakHis) and Invasive Ductal Carcinoma (IDC). However, the study also states that no single model significantly outperformed existing methods, indicating the need for further architectural improvements. Graph-based approaches have also gained traction for multi-class classification tasks. Wang et al. [16] proposed a connectivity-aware graph transformer (CGT) that models biological entities as graph nodes and their spatial relationships as edges. By combining this connectivity information with the attention mechanism, CGT can effectively capture local and global dependencies.

Similarly, SCUBa-Net [17] introduces a bi-graph interaction network that combines constrained and unconstrained graphs. The model leverages both local structural information and global context through inter-graph and intra-graph interactions, which demonstrates superior performance compared to traditional CNN and transformer-based models. Inspired by the diagnostic strategies of pathologists, Li et al. [18] developed a tree-like framework named DeepTree. Instead of treating all classes differently, DeepTree models hierarchical relationships between tissue morphologies, enabling better interpretability and performance. This approach highlights the importance of integrating clinical reasoning into deep learning models.

Recently, HER2 classification from immunohistochemistry (IHC) images has gained considerable attention due to its critical role in treatment planning and prognosis. HER2 IHC scoring uses four categories (0, 1+, 2+, and 3+). Diverse staining patterns, inconsistent tissue architecture, and multi-scale morphological characteristics define the complexity of histopathology images. These characteristics pose major obstacles to conventional deep learning models. As a result, recent research has focused on improving feature representation using attention-based mechanisms, multi-scale feature extraction strategies, and hybrid architectures, which aim to better capture local and global information.

A deep learning framework combining CNN and DenseNet121 with Grad-CAM and SHAP for interpretability has been proposed by Nabi et al. [13]. These techniques provide explanations for model decisions, enhancing clinical trust. This model achieved high accuracy while addressing the “black-box” nature of deep learning, which is a critical requirement in medical applications. Nabi et al. [19] introduced a hybrid architecture to improve HER2 scoring. A combination of ResNet-18 and EfficientNet-B0 demonstrated strong performance by leveraging both spatial and feature extraction. Such hybrid models are particularly effective in distinguishing borderline cases (e.g., 1+ vs. 2+), which are often challenging even for expert pathologists.

## 3. Methodology

**Figure 1:**
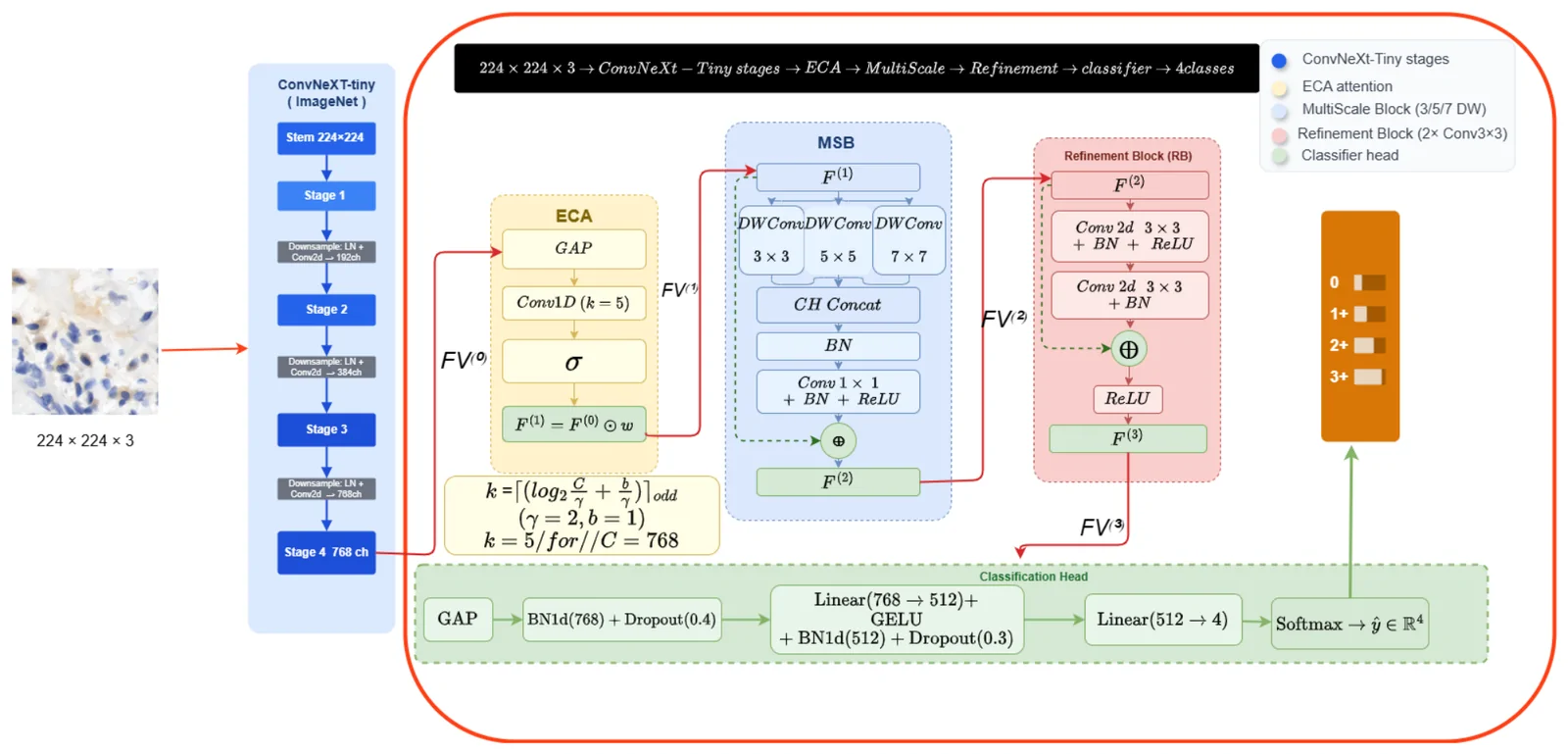
Proposed Multi-scale Local Feature Refinement Network architecture.

We propose a deep learning framework, termed the Multi-scale Local Feature Refinement Network (MLFRNet), for histopathology image classification. Let the dataset be $D=\{\left(x_{i},y_{i}\right)\}$, where $x_{i}\in R^{W\times H\times 3}$ represents a histopathology image and $y_{i}\in \left\{1,2,\ldots ,C_{\mathrm{class}}\right\}$ denotes its class label. The objective is to learn a parametric function $f\left(\theta \right):R^{W\times H\times 3}\to R^{C}$, parameterized by θ, that maps each input image to one of *C* classes. The optimal parameters $\theta^{*}$ are obtained by minimizing the composite loss $L_{\mathrm{total}}$. The proposed framework consists of the ConvNeXt-Tiny backbone, the multi-scale feature-refinement block, and the classification block, as shown in Figure 1. The details of each component are described below.

### 3.1. Preprocessing

The input images were high-resolution breast cancer biopsy patches acquired at 40× optical magnification, with a size of 1024×1024 pixels in RGB format. During training, each patch was processed by an augmentation pipeline to improve the robustness of the model to variations in tissue appearance, biopsy orientation, staining, and image acquisition. First, a random region covering 80–100% of the original image area was selected with an aspect ratio ranging from 3/4 to 4/3 and resized to 224×224 pixels using bicubic interpolation. The 224×224 input size was selected to match the input requirements of the pretrained ConvNeXt-Tiny backbone while retaining sufficient cellular and membrane-related information for HER2 classification and maintaining reasonable computational requirements. Random horizontal and vertical flipping was applied with a probability of 0.5, and random rotation within the range of [−15°,+15°] was used to account for variations in biopsy orientation. To improve robustness to differences in staining and image acquisition, moderate augmentation was applied by varying brightness, contrast, saturation, and hue with magnitudes of 0.15, 0.15, 0.15, and 0.05, respectively. After augmentation, the pixel values were scaled to the range [0,1] and normalized channel-wise using the ImageNet mean μ=[0.485,0.456,0.406] and standard deviation σ=[0.229,0.224,0.225]. For each color channel c∈{R,G,B}, the normalized pixel value was calculated as ${\tilde{x}}_{c}=\left(x_{c}-\mu_{c}\right)/\sigma_{c}$. This preprocessing strategy was used to provide consistent input to the pretrained network while preserving the morphological information relevant to HER2 score classification. The description above reflects the preprocessing implemented for the reported experiments.

### 3.2. Feature Extraction Using ConvNeXt-Tiny

This study utilized a pre-trained ConvNeXt-Tiny model as the feature-extraction backbone. ConvNeXt-Tiny is a modernized convolutional architecture that incorporates design principles from Vision Transformers while retaining the spatial inductive bias of conventional CNNs. These include large 7×7 depthwise separable convolutions, increased channel expansion ratios, Gaussian Error Linear Unit (GELU) activation, and a simplified normalization design. The ConvNeXt-Tiny backbone contains four hierarchical stages with channel dimensions of 96, 192, 384, and 768, respectively. Given a standardized input image $X\in R^{3\times 224\times 224}$, the backbone progressively reduces the spatial resolution through a cumulative stride of 32 while increasing the feature dimension. The resulting feature representation is obtained as $FV^{\left(0\right)}=f_{\theta }\left(X\right)\in R^{768\times 7\times 7}$. The backbone is initialized with ImageNet-1K pretrained weights [20,21].

### 3.3. Proposed Multi-Scale Local Feature Refinement Block

The proposed study is formulated as a sequence of transformation blocks, with the overall model expressed as a hierarchical mapping. The complete pipeline is represented in Equation (1).

(1) $$M\left(x\right)=x\overset{F_{\theta }}{\to }\overset{\mathrm{ECA}}{\to }\overset{\mathrm{MSB}}{\to }\overset{\mathrm{RB}}{\to }\overset{\mathrm{Classifier}}{\to }\hat{y}$$

This formulation represents a structured architectural design in which each component performs a specific transformation within the feature space. For histopathology image analysis, these transformations facilitate the extraction and refinement of complementary spatial and contextual features. The details of each transformation block are described below.

#### 3.3.1. Efficient Channel Attention (ECA) Block

The attention mechanism can enhance feature representation by reweighting learned responses according to their estimated relevance. Efficient Channel Attention (ECA) [22] was proposed to avoid dimensionality reduction and uses one-dimensional convolution to capture channels. This design avoids an explicit dimensionality-reduction step while providing channel-wise feature recalibration. Thus, we have utilized an ECA block as the initial step for feature enhancement through channel attention. Following the feature extraction stage, the obtained representation $FV^{\left(0\right)}\in R^{768\times 7\times 7}$ is further refined using the ECA block. This allows the network to emphasize informative features by using channel-wise weights.

Formally, given the input feature tensor $FV^{\left(0\right)}={\left\{f_{c}^{\left(0\right)}\right\}}_{c=1}^{C}$, where C=768 represents the number of channels and each $f_{c}^{\left(0\right)}\in R^{H\times W}$ corresponds to a spatial feature map with H=W=7, the ECA transformation produces $FV^{\left(1\right)}\in R^{768\times 7\times 7}$ as given in Equation (2).

(2) $$FV^{\left(1\right)}=\mathrm{ECA}\left(FV^{\left(0\right)}\right)={\left\{w_{c}\cdot f_{c}^{\left(0\right)}\right\}}_{c=1}^{C}$$

where $w_{c}\in R$ represents the attention weight corresponding to the *c*-th channel. This ensures that each feature map will correspond to its importance coefficient. A detailed algorithm for implementation of an ECA block is represented in Algorithm 1. The process begins with a global spatial aggregation that compresses each channel into a scalar. Specifically, for each channel c∈{1,2,…,C}, a global descriptor $z_{c}$ is obtained using global average pooling. To model inter-channel dependencies, *z* is processed using a one-dimensional convolutional operator with the kernel size *k*. The convolutional operator is later activated by the sigmoid function σ to get the channel weights $w\in \{w_{1},w_{2},\ldots w_{c}\}$.
**Algorithm 1:** Efficient Channel Attention (ECA)**Input:** $FV^{\left(0\right)}$**Output:** $FV^{\left(1\right)}$$z\leftarrow \mathrm{GAP}(FV^{\left(0\right)})$;$k\leftarrow {\left|\frac{\log_{2}\left(C\right)}{\gamma }+\frac{b}{\gamma }\right|}_{\mathrm{odd}}$, with γ=2, b=1 (yielding k=5 for C=768);w←σ(Conv1D(z;k));w←reshape(w,B×C×1×1);$FV^{\left(1\right)}\leftarrow FV^{\left(0\right)}⊙w$;**return** $FV^{\left(1\right)}$;

Finally, the transformed feature vector $FV^{\left(1\right)}$ is obtained with the dot product of channel weights and the previous feature vector. This transformation preserves the original dimensionality, ensuring that the output feature tensor remains $FV^{\left(1\right)}\in R^{768\times 7\times 7}$.

#### 3.3.2. Multi-Scale Block (MSB)

The ECA block performs channel-wise feature recalibration but does not explicitly model spatial relationships within the feature maps. In histopathology images, relevant visual information can occur across different spatial neighborhood extents. To complement the channel-wise processing of ECA, the proposed Multi-scale Block (MSB) employs parallel depthwise convolutions with different kernel sizes to capture complementary spatial information and enhance multi-scale feature representation.

The input to the MSB is the feature representation $FV^{\left(1\right)}\in R^{C\times H\times W}$ produced by the ECA block. In the proposed architecture, C=768 and H=W=7, such that $FV^{\left(1\right)}\in R^{768\times 7\times 7}$. Importantly, the three depthwise convolution branches operate on this same 768×7×7 feature representation rather than on feature maps corresponding to different stages or resolutions. The branches use 3×3, 5×5, and 7×7 kernels, respectively, and their outputs can be expressed as $F_{k}=\psi_{k}\left(FV^{\left(1\right)}\right)$, where k∈{3,5,7} and $\psi_{k}\left(\cdot \right)$ denotes a depthwise convolution with a k×k kernel. Since all three branches receive the same input feature map and preserve the channel dimension, the different kernels provide different spatial neighborhood extents within the same feature representation. These Convolution branch operations and feature-fusion are summarized in Algorithm 2.
**Algorithm 2:** Multi-Scale Depthwise Convolution Block (MSB)**Input:** $FV^{\left(1\right)}$**Output:** $FV^{\left(2\right)}$$F_{3}\leftarrow {\mathrm{DWConv}}_{3\times 3}\left(FV^{\left(1\right)}\right)$;$F_{5}\leftarrow {\mathrm{DWConv}}_{5\times 5}\left(FV^{\left(1\right)}\right)$;$F_{7}\leftarrow {\mathrm{DWConv}}_{7\times 7}\left(FV^{\left(1\right)}\right)$;$F_{\mathrm{concat}}\leftarrow \mathrm{Concat}\left(F_{3},F_{5},F_{7}\right)$;$F_{\mathrm{norm}}\leftarrow \mathrm{BN}\left(F_{\mathrm{concat}}\right)$;$F_{\mathrm{proj}}\leftarrow {\mathrm{Conv}}_{1\times 1}\left(F_{\mathrm{norm}}\right)$;$F_{\mathrm{fused}}\leftarrow \mathrm{ReLU}\left(\mathrm{BN}\left(F_{\mathrm{proj}}\right)\right)$;$FV^{\left(2\right)}\leftarrow F_{\mathrm{fused}}⊕FV^{\left(1\right)}$;**return** $FV^{\left(2\right)}$;

Specifically, the 3×3 branch processes a smaller local neighborhood, the 5×5 branch incorporates a larger neighborhood, and the 7×7 branch provides the largest neighborhood among the three branches. These kernel sizes therefore define different convolutional spatial contexts within the final 7×7 feature map. They are not interpreted as direct representations of cellular, cell-cluster, or tissue-level structures. Rather, the biological motivation for using multiple kernels arises from the presence of visual patterns with varying spatial extents in histopathological images, while the individual kernel-to-biological-scale correspondence is not assumed.

The outputs of the three depthwise convolution branches are subsequently concatenated along the channel dimension as $F_{\mathrm{concat}}=\mathrm{Concat}\left(F_{3},F_{5},F_{7}\right)$, resulting in $F_{\mathrm{concat}}\in R^{3C\times H\times W}$. Batch normalization is then applied to the concatenated representation, giving $F_{\mathrm{norm}}=\mathrm{BN}\left(F_{\mathrm{concat}}\right)$. A 1×1 convolution $\psi_{1}$ is subsequently used to project the 3C channels back to the original channel dimension, expressed as $F_{\mathrm{proj}}=\psi_{1}\left(F_{\mathrm{norm}}\right)$. The projected representation is then normalized and activated using ReLU, yielding $F_{\mathrm{fused}}=\mathrm{ReLU}(\mathrm{BN}\left(F_{\mathrm{proj}}\right))$.

Finally, a residual connection combines the transformed multi-scale representation with the original input feature map, producing the MSB output as $FV^{\left(2\right)}=F_{\mathrm{fused}}⊕FV^{\left(1\right)}$. Through this parallel design, the MSB combines complementary spatial information obtained from multiple kernel sizes while maintaining the original feature dimension. Thus, the 3×3, 5×5, and 7×7 convolutions should be understood as providing different spatial neighborhood extents within a common feature representation, rather than as explicitly encoding predefined biological scales. The resulting multi-scale representation is subsequently passed to the Refinement Block for further feature refinement.

#### 3.3.3. Refinement Block

The feature space $FV^{\left(2\right)}\in R^{C\times H\times W}$ (C=768, H=7, W=7) is further processed using the Refinement Block (RB), as shown in Algorithm 3. The RB is designed to refine the feature representation. In histopathology images, staining variations and acquisition artifacts are common; therefore, feature refinement may improve the robustness of the learned representation. The Refinement Block consists of a two-stage convolutional refinement process. First, the input feature map undergoes a 3×3 convolution followed by batch normalization and ReLU activation. This is followed by a second 3×3 convolution and batch normalization. All convolutions in RB use stride 1, padding 1 (preserving the spatial size), and no channel grouping. RB performs residual feature enhancement, in which the learned transformations $H_{1}$ and $H_{2}$ refine feature responses while preserving the input representation. This design is intended to improve the processing of diagnostically relevant features that may be affected by acquisition artifacts.
**Algorithm 3:** Refinement Block (RB)**Input:** $FV^{\left(2\right)}$**Output:** $FV^{\left(3\right)}$$H_{1}\left(FV^{\left(2\right)}\right)\leftarrow \mathrm{ReLU}\left(\mathrm{BN}({\mathrm{Conv}}_{3\times 3}\left(FV^{\left(2\right)}\right))\right)$;$H_{2}\left(FV^{\left(2\right)}\right)\leftarrow \mathrm{BN}\left({\mathrm{Conv}}_{3\times 3}\left(H_{1}\left(FV^{\left(2\right)}\right)\right)\right)$;$FV^{\left(3\right)}\leftarrow \mathrm{ReLU}\left(H_{2}\left(FV^{\left(2\right)}\right)+FV^{\left(2\right)}\right)$;**return** $FV^{\left(3\right)}$;

### 3.4. Classification Block

Following the RB, the feature map $FV^{\left(3\right)}$ is transformed into a semantically meaningful one through a global aggregation. Specifically, global average pooling is employed to collapse the spatial dimension by converting the feature map into the channel-wise descriptor. This operation computes the average activations across spatial locations for each channel, and the resulting feature vector will be $f=\{f_{1},f_{2},\ldots ,f_{C}\}$. This process is represented as follows:

(3) $$f_{c}=\frac{1}{HW}\sum_{i=1}^{H}\sum_{j=1}^{W}FV_{c}^{\left(3\right)}\left(i,j\right),\;c\in \left\{1,\ldots ,C\right\}.$$

The pooled descriptor is then processed by the classification head through a sequence of normalization, regularization, nonlinear projection, and classification operations. Specifically, f is first normalized using Batch normalization to obtain $h_{0}={\mathrm{BN}}_{1}\left(f\right)$, followed by a dropout layer with rate 0.4 producing $h_{1}={\mathrm{Dropout}}_{0.4}\left(h_{0}\right)$. The resulting representation is then projected through a linear layer $W_{1}\in R^{512\times 768}$ with bias $b_{1}$ and GELU activation δ(·), giving $h_{2}=\delta \left(W_{1}h_{1}+b_{1}\right)$. This representation is subsequently normalized by a second batch Normalization layer, $h_{3}={\mathrm{BN}}_{2}\left(h_{2}\right)$, and regularized using a second dropout layer with rate 0.3, yielding $h_{4}={\mathrm{Dropout}}_{0.3}\left(h_{3}\right)$. Finally, the resulting representation is mapped to the *K* output classes through a linear projection $W_{2}\in R^{K\times 512}$ with bias $b_{2}$, followed by Softmax:

(4) $$\hat{y}=\mathrm{Softmax}\left(W_{2}h_{4}+b_{2}\right)\in R^{K}.$$

Here, K=4 denotes the four HER2 score classes.

### 3.5. Loss Computation and Optimization

Algorithm 4 details the complete training process of MLFRNet, including loss computation. The feature maps obtained at each stage are summarized in Table 1. Each batch is passed sequentially through ConvNeXt-Tiny, ECA, MSB, and RB to produce $FV^{\left(0\right)}$, $FV^{\left(1\right)}$, $FV^{\left(2\right)}$, and $FV^{\left(3\right)}$, respectively. Batch normalization stabilizes the feature distribution, followed by dropout, which introduces regularization by randomly suppressing activations and thereby reducing overfitting. The first linear projection $W_{1}$ maps the pooled feature vector into a lower-dimensional latent space, where the nonlinear activation enables complex feature interactions. The second projection $W_{2}$ maps this representation to the *K* output classes. The classifier produces logits z followed by a softmax mapping to the predicted probability vector $\hat{y}$. The training objective combines label-smoothed cross-entropy with focal loss [23]. Let ${\hat{y}}_{i}$ denote the predicted probability vector for sample *i* and $y_{i}$ the one-hot ground-truth label. Label smoothing with parameter ϵ=0.1 redistributes a small probability mass across the *K* classes:

(5) $$y_{i}^{LS}=\left(1-\epsilon \right)y_{i}+\frac{\epsilon }{K}.$$

The label-smoothed cross-entropy loss is defined over a batch of *B* samples as

(6) $$L_{\mathrm{CE}-\mathrm{LS}}=-\frac{1}{B}\sum_{i=1}^{B}\sum_{k=1}^{K}y_{i,k}^{LS}\log\left({\hat{y}}_{i,k}\right),$$

where $y_{i,k}^{LS}$ is defined in Equation (5). The focal-loss term is evaluated using the predicted probability $p_{t,i}$ assigned to the true class of sample *i*:

(7) $$L_{\mathrm{Focal}}=-\frac{\alpha }{B}\sum_{i=1}^{B}{\left(1-p_{t,i}\right)}^{\gamma }\log\left(p_{t,i}\right),$$

with class-weight parameter α=1 and focusing parameter γ=2. Thus, no class-specific reweighting is introduced by α in the present experiments. The final composite training objective is

(8) $$L_{\mathrm{total}}=L_{\mathrm{CE}-\mathrm{LS}}+\lambda L_{\mathrm{Focal}},\;Where\;\lambda =0.15.$$

These parameter values (ϵ=0.1, α=1, γ=2, and λ=0.15) are used consistently in the methodology and Algorithm 4. $L_{\mathrm{total}}$ is backpropagated to fine-tune all model parameters $\theta =\{\theta_{\mathrm{backbone}},\theta_{\mathrm{ECA}},\theta_{\mathrm{MSB}},\theta_{\mathrm{RB}},\theta_{\mathrm{cls}}\}$.

Optimization is performed using AdamW with an initial learning rate of $2.5\times 10^{-4}$, $\beta_{1}=0.9$, $\beta_{2}=0.999$, and weight decay of $5\times 10^{-5}$. A OneCycleLR policy with cosine annealing is used over 15 epochs, with a maximum learning rate of $4.0\times 10^{-4}$ and 25% of the schedule used for the increasing-learning-rate phase. The test set is not used for scheduler selection or checkpoint selection. The exact selection procedure is specified in Algorithm 4.
**Algorithm 4:** Overall Training Process of MLFRNet**Input:** Dataset $D={\left\{\left(x_{i},y_{i}\right)\right\}}_{i=1}^{N}$, K=4, batch size B=32, and $N_{e}=15$ epochs**Output:** Selected MLFRNet model and predicted class label $\hat{y}$X←Preprocessing(X);Initialize $\theta =\{\theta_{\mathrm{backbone}},\theta_{\mathrm{ECA}},\theta_{\mathrm{MSB}},\theta_{\mathrm{RB}},\theta_{\mathrm{cls}}\}$;Initialize AdamW: $\eta_{0}=2.5\times 10^{-4}$, $\left(\beta_{1},\beta_{2}\right)=\left(0.9,0.999\right)$, $\omega =5\times 10^{-5}$;Initialize OneCycleLR: $\eta_{\max}=4.0\times 10^{-4}$, $q_{\mathrm{warm}}=0.25$, cosine annealing;
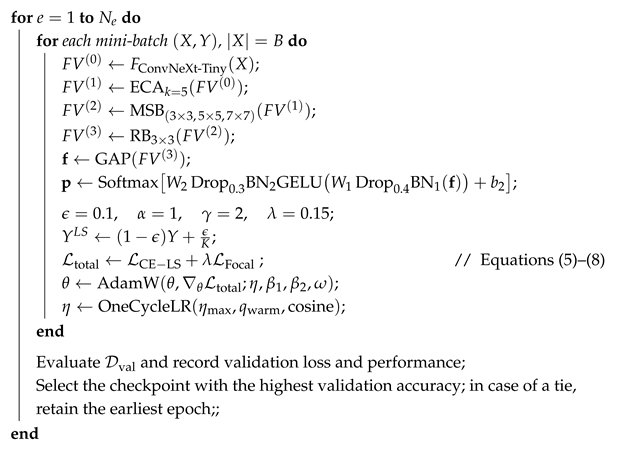
**Test:** $D_{\mathrm{test}}$ is excluded from optimization, checkpoint selection, and training-duration decisions;For an evaluation sample, $\hat{y}\leftarrow \arg\max_{k}p_{k}$;**return** Selected MLFRNet model and $\hat{y}$;

## 4. Analysis of Results

The proposed Multi-scale Local Feature Refinement Network (MLFRNet) with Channel Attention and Multi-scale Depthwise Convolution is evaluated on the HER2-IHC-40× dataset. This section presents the quantitative and qualitative evaluation of MLFRNet, including the ablation studies, classification analysis, visualization analyses, and computational characterization.

### 4.1. Dataset Details

The evaluation of MLFRNet was performed using the publicly available HER2-IHC-40× benchmark dataset introduced by Nabi et al. [24]. The dataset contains 10,997 standardized histopathological image patches obtained from surgical breast cancer biopsy specimens stained using standard HER2 immunohistochemical protocols and digitized at 40× optical magnification at the University of Malaya Medical Centre (UMMC), Kuala Lumpur, Malaysia. Each image patch has a native resolution of 1024×1024 pixels. The images are assigned to one of four standard ASCO/CAP diagnostic categories: Score 0, Score 1+, Score 2+, and Score 3+, according to the annotations provided by expert surgical pathologists. The benchmark dataset is divided into a predefined training pool containing 8797 patches and an official test partition containing 2200 patches. The predefined development pool of 8797 patches was divided into a training subset of 7037 patches and an internal validation subset of 1760 patches using an 80/20 stratified patch-level split. The three reported runs used seeds 42, 142, and 242. Within each seed, the same training/validation partition was used for all compared configurations. Model parameters were optimized using the training subset, while checkpoint selection and model-selection decisions were based on validation performance. The fixed official test partition of 2200 patches was used to calculate performance metrics for each evaluated baseline, loss formulation, architectural ablation, and input-resolution experiment. The test partition was not used for gradient updates, checkpoint selection, hyperparameter tuning, or training-duration decisions. Test-set results were used to report the performance of the evaluated configurations.

The class distribution of the training pool and test set is presented in Table 2. The publicly released HER2-IHC-40× dataset provides pre-extracted image patches organized into four diagnostic classes. However, the public release does not provide patient identifiers, biopsy accession identifiers, or whole-slide image source coordinates. Consequently, the independence of the partitions at the patient and slide levels cannot be independently verified using the available data, and re-splitting the dataset to ensure patient-level or slide-level separation is not possible. Accordingly, the manuscript does not claim patient- or slide-disjoint evaluation. To address one identifiable source of potential overlap, we performed an image-content audit comparing all 8797 development images with all 2200 test images. The audit found 0 exact byte-level duplicates using SHA-256 hashing and 0 perceptual-hash candidate pairs under the predefined pHash/dHash screening threshold; no candidate pair exceeded the SSIM (Structural Similarity Index Measure) flag threshold. A complementary ConvNeXt-Tiny embedding audit gave a maximum nearest-neighbor cosine similarity of 0.9846 and identified 10 pairs above 0.98; these were treated as similarity flags rather than duplicates. The audit addresses image-content overlap only and does not establish patient- or whole-slide-level independence. The pHash and dHash screening thresholds were set to a Hamming distance of ≤10/64 bits. Candidate similarity was assessed using grayscale SSIM after deterministic resizing to 256×256 pixels, with SSIM ≥0.90 used as the flag threshold. The frozen ImageNet-pretrained ConvNeXt-Tiny embedding audit used 224×224 inputs and L2-normalized 768-dimensional pooled features; 10 test/development pairs exceeded cosine similarity of 0.98. All ten flagged pairs were visually reviewed and were visually distinct, showing similar broad histopathological morphology but different cellular or spatial arrangements; they were therefore retained as similarity flags rather than classified as duplicates. The pHash/dHash procedure used a nearest-neighbor candidate search rather than an exhaustive enumeration of every possible qualifying cross-partition pair. A summary of the ten flagged pairs and their visual assessments is provided in Supplementary Table S2, with the corresponding pairwise images shown in Supplementary Figure S1.

### 4.2. Experimental Setup

The proposed study was implemented in PyTorch2.11.0+cu128. Experiments were run on an NVIDIA Tesla T4 GPU with 16 GB of GPU memory. Prior to model training and evaluation, each image was resized to a fixed resolution of 224×224 pixels for the primary experiments. During training, random resized cropping, horizontal and vertical flipping, rotation (±15°), and color jittering were applied. For validation and testing, the deterministic transform consisted of resizing to 224×224 pixels, conversion to a tensor (scaling pixel values to [0,1]), and channel-wise ImageNet normalization with mean (0.485,0.456,0.406) and standard deviation (0.229,0.224,0.225). A batch size of 32 was used for the primary 224×224 experiments. Model parameters were optimized for up to 15 epochs using AdamW with learning rate $2.5\times 10^{-4}$, weight decay $5\times 10^{-5}$, and AdamW moment coefficients $\beta_{1}=0.9$ and $\beta_{2}=0.999$. A OneCycleLR scheduler was used with a maximum learning rate of $4.0\times 10^{-4}$ and a 25% increasing-learning-rate phase. The composite loss combined label-smoothed cross-entropy (ϵ=0.1) with focal loss (α=1, γ=2, λ=0.15). During testing, no augmentations were applied. For the reported experiments, the maximum training schedule was fixed at 15 epochs, and checkpoint selection within this schedule was based on validation accuracy, as specified in Algorithm 4. For the Seed-42 run, validation accuracy reached 95.45% at epoch 13, and epochs 14–15 did not exceed this value. An additional extended-training analysis to 50 epochs was performed only as a post hoc descriptive check after the reported training protocol had been fixed. The fixed official test partition was used to evaluate the selected checkpoint for each experimental configuration and calculate the corresponding performance metrics. However, test-set results were not used for gradient-based training, checkpoint selection, hyperparameter tuning, or training-duration decisions, and model-selection decisions were based on validation performance.

### 4.3. Ablation Study

ConvNeXt-Tiny was selected as the backbone after the internal baseline comparison. We then evaluated alternative loss formulations and feature-refinement configurations using the same experimental framework. The architectural ablations were repeated for the three prespecified seeds (42,142, and 242) to characterize run-to-run variability.

- **With different loss functions:** Cross-entropy (CE) loss is the commonly used loss function for the multi-class classification. The HER2-IHC-40× dataset exhibits an imbalanced class distribution, particularly for Class 2+. Therefore, we evaluated different loss formulations to improve robustness to difficult samples and overall classification performance.
  Focal loss [23] was employed to place greater emphasis on difficult and misclassified samples, which can be beneficial in the presence of class imbalance. The focal-loss focusing parameter was set to γ=2 with α=1, meaning that no additional class-specific weighting was applied. Label smoothing (LS) was also employed to improve the generalization ability of the model.
  Table 3 presents the mean performance of the ConvNeXt-Tiny model under different loss formulations, including cross-entropy (CE), focal loss, label smoothing, and their combination. Among the evaluated formulations, the proposed composite loss achieved the highest observed mean test accuracy of 94.24%. It combines label-smoothed cross-entropy with focal loss using a label-smoothing factor of 0.1 and a focal-loss contribution weight of 0.15. The training pool contains 905 Class 2+ patches out of 8797 patches (10.29%), compared with Class 0 (3031), Class 1+ (2151), and Class 3+ (2710) patches. Although this distribution is imbalanced, explicit class-dependent loss weights were not introduced in this ablation as the purpose was to isolate the effects of focal modulation and label smoothing. Thus, α=1 should not be interpreted as an imbalance-correction weight. The class imbalance is acknowledged as a limitation of the present loss design. All rows in Table 3 were evaluated using the same fixed official test partition. The test-set results provide a descriptive comparison of the evaluated loss formulations; the loss formulation used in the final model was determined through the validation-based model-selection procedure, rather than by selecting the formulation with the highest test accuracy.
- **With different feature refinement blocks:**
  The proposed multi-scale local feature refinement architecture was evaluated through an ablation study to examine the empirical effects of each block. All configurations were implemented using the same ConvNeXt-Tiny backbone, adaptive average pooling, and classification head while varying the blocks. As shown in Figure 1, the proposed architecture follows a sequential ECA → MSB → RB pathway.

**Table 3:** Ablation study results of ConvNeXt-Tiny with different loss formulations. Mean ± SD values are final evaluations on the official 2200-patch test partition across the three prespecified runs; loss formulation and checkpoint selection were performed during model development using the validation subset.

Loss Function	Accuracy (%)	F1-Score (%)
CE loss	93.09 ± 0.57	91.82 ± 0.48
Focal Loss	92.88 ± 0.25	91.59 ± 0.28
CE + LS	93.38 ± 0.17	92.23 ± 0.51
CE + LS + Focal	94.24 ± 1.78	93.12 ± 2.07

All three-run mean ± SD summaries reported in the ablation and baseline-comparison tables were checked against the corresponding seed-wise evaluation records and use the sample standard deviation. The individual-seed accuracy and macro-F1 values underlying the loss and architectural ablations are provided in Supplementary Table S1B.

The ablation results in Table 4 show that model performance depends on the configuration of the feature-refinement blocks. The baseline ConvNeXt-Tiny achieves 94.24% accuracy and 93.12% F1-score. Adding ECA alone improves the results to 94.76% and 93.72%, respectively, indicating a modest benefit from channel-wise feature recalibration. MSB provides the largest observed improvement among the individual blocks, achieving 95.68% accuracy and 94.70% F1-score, while RB achieves 95.53% accuracy and 94.58% F1-score.

For the two-block configurations, ECA + MSB achieves 94.69% Accuracy and 93.84% F1-score, which is lower than MSB alone. In contrast, ECA + RB achieves 95.68% Accuracy and 94.66% F1-score, showing a small improvement over RB alone. The MSB + RB configuration provides the strongest two-block performance, with 95.92% Accuracy and a 95.08% F1-score. These results indicate that the contribution of each block is configuration dependent rather than uniformly additive.

The complete ECA + MSB + RB configuration has the highest observed mean among the evaluated configurations, with 96.10% accuracy and 95.30% F1-score. Relative to the plain ConvNeXt-Tiny baseline, the mean differences are 1.86 and 2.18 percentage points, respectively. The difference from MSB + RB is smaller, and the three-seed standard deviations indicate appreciable run-to-run variation. We therefore describe the complete model as the highest observed mean in this ablation set rather than as a statistically established improvement. ECA is retained as part of the final architecture because its channel-recalibration operation complements the spatial processing of MSB and RB while adding only a small number of parameters (Table 5).

The reduced-kernel experiments show that the (3,7) configuration has the highest observed mean among the evaluated two-kernel variants, achieving 95.98% accuracy and a 95.01% F1-score. However, the complete architecture using all three MSB kernels achieves higher performance. This supports retaining the standard 3×3, 5×5, and 7×7 multi-scale design in the final model.

### 4.4. Performance of the Proposed MLFRNet Model

Table 6 presents the performance of MLFRNet over three prespecified seeds (42, 142, and 242) using the 2200-patch official test partition. The three seeds were fixed before final evaluation to characterize run-to-run variability under the specified experimental protocol. As the training/validation partition was regenerated for each seed, the observed between-run variation reflects both training stochasticity and variation in the corresponding training/validation partition. Within each seed, all compared configurations used the same corresponding partition, enabling paired comparisons. The official test partition was kept separate throughout model development and was not used for architecture selection, loss-function selection, hyperparameter tuning, checkpoint selection, or training-duration decisions. It was evaluated only after the model-development stage had been completed. Thus, none of the three seeds was chosen on the basis of test performance.

For a metric *M*, the reported value is the mean and standard deviation obtained from the three runs. The mean is given by

$$\bar{M}=\frac{1}{S}\sum_{s=1}^{S}M_{s}$$

where S=3 is the number of runs and $M_{s}$ represents the metric obtained for seed *s*. The sample standard deviation is calculated as

$$SD\left(M\right)=\sqrt{\frac{1}{S-1}\sum_{s=1}^{S}{\left(M_{s}-\bar{M}\right)}^{2}}.$$

The mean of the three runs is used as the main estimate rather than selecting the best-performing seed. Across the three runs, MLFRNet obtained an accuracy of 96.10±0.22%, precision of 95.34±0.18%, recall of 95.29±0.34%, F1-score of 95.30±0.23%, and AUROC of 99.57±0.05%. The reported standard deviations describe between-run variation under these seed-specific experimental partitions and should not be interpreted as uncertainty estimates for generalization to new patients or whole-slide specimens. The results for each seed are also included in Table 6 for reproducibility.

### 4.5. Computational Characterization

To characterize the computational requirements of the proposed MLFRNet model, we measured trainable parameters, multiply-accumulate operations (MACs), approximate floating-point operations (FLOPs), serialized model-file size, inference latency, throughput, and peak GPU memory consumption. All measurements were obtained using an NVIDIA Tesla T4 GPU with an input resolution of 224×224 pixels. MACs were measured using the PyTorch-OpCounter (THOP) library, and FLOPs (Floating-Point Operations) were estimated using the conventional approximation FLOPs≈2×MACs. Inference latency was measured after 20 warm-up iterations over 100 timed iterations using CUDA (Compute Unified Device Architecture) events for batch sizes of 1 and 32. Throughput was calculated from the corresponding batch size and mean inference latency. Peak GPU memory consumption was measured during batch-32 inference. The profiling environment used PyTorch 2.11.0+cu128, torchvision 0.26.0+cu128, and the THOP profiling implementation. These measurements characterize the selected MLFRNet checkpoint at 224×224 input resolution and are not used as evidence of computational superiority over competing architectures.

The total number of trainable parameters is calculated as

(9) $$P=\sum_{l=1}^{L}P_{l},$$

where $P_{l}$ denotes the number of trainable parameters in the *l*-th layer. The reported floating-point operation count is estimated from the measured MACs as

(10) FLOPs≈2×MACs.

For *N* timed inference iterations, the mean latency is calculated as

(11) $$\bar{T}=\frac{1}{N}\sum_{i=1}^{N}T_{i},$$

where $T_{i}$ is the inference time of the *i*-th iteration. The corresponding throughput is

(12) $$\mathrm{Throughput}=\frac{B}{\bar{T}/1000},$$

where *B* denotes the inference batch size and $\bar{T}$ is the mean latency in milliseconds.

Table 7 summarizes the computational characteristics of MLFRNet. The model contains 41.447 million trainable parameters and requires 18.465 G MACs, corresponding to approximately 36.930 G FLOPs under the conventional two-FLOP-per-MAC approximation. The serialized model file size is 158.28 MB. On an NVIDIA Tesla T4 GPU, the measured inference latency was 12.421±4.258 ms for batch size 1 and 134.128±1.509 ms for batch size 32, corresponding to throughputs of 80.5 and 238.6 images/s, respectively. Peak GPU memory consumption was 920.4 MB allocated and 1145.0 MB reserved. These measurements provide a computational profile of the proposed model and are not interpreted as evidence of computational superiority over other architectures.

**Figure 2:**
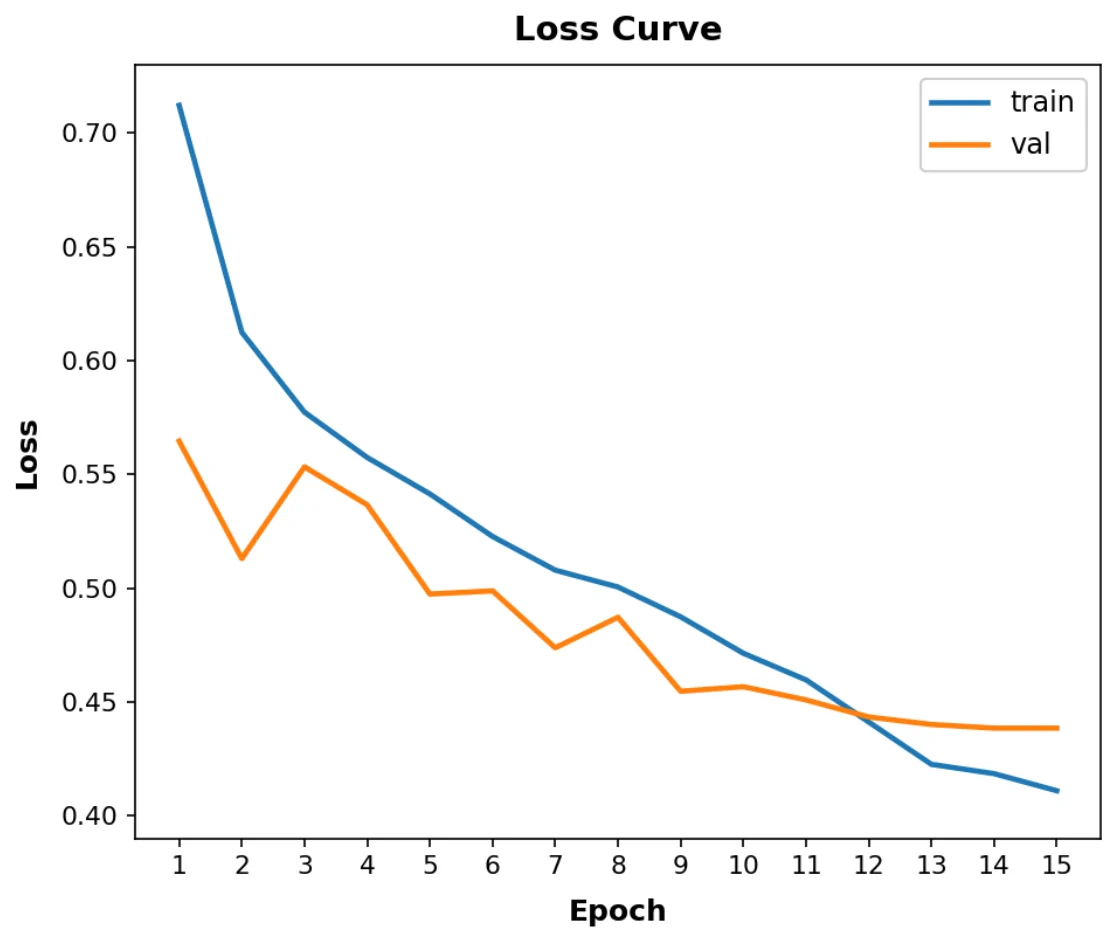
Training and validation loss curves of the proposed MLFRNet model over 15 epochs.

The training and validation loss curves are shown in Figure 2. For the seed-42 run, validation accuracy reached 95.45% at epoch 13 and did not improve at epochs 14 or 15. Although the training loss continued to decrease, the validation accuracy did not show a sustained improvement during the final epochs. The checkpoint was selected using validation accuracy according to Algorithm 4, with ties retaining the earlier checkpoint. The test-partition results were not used in this selection.

**Figure 3:**
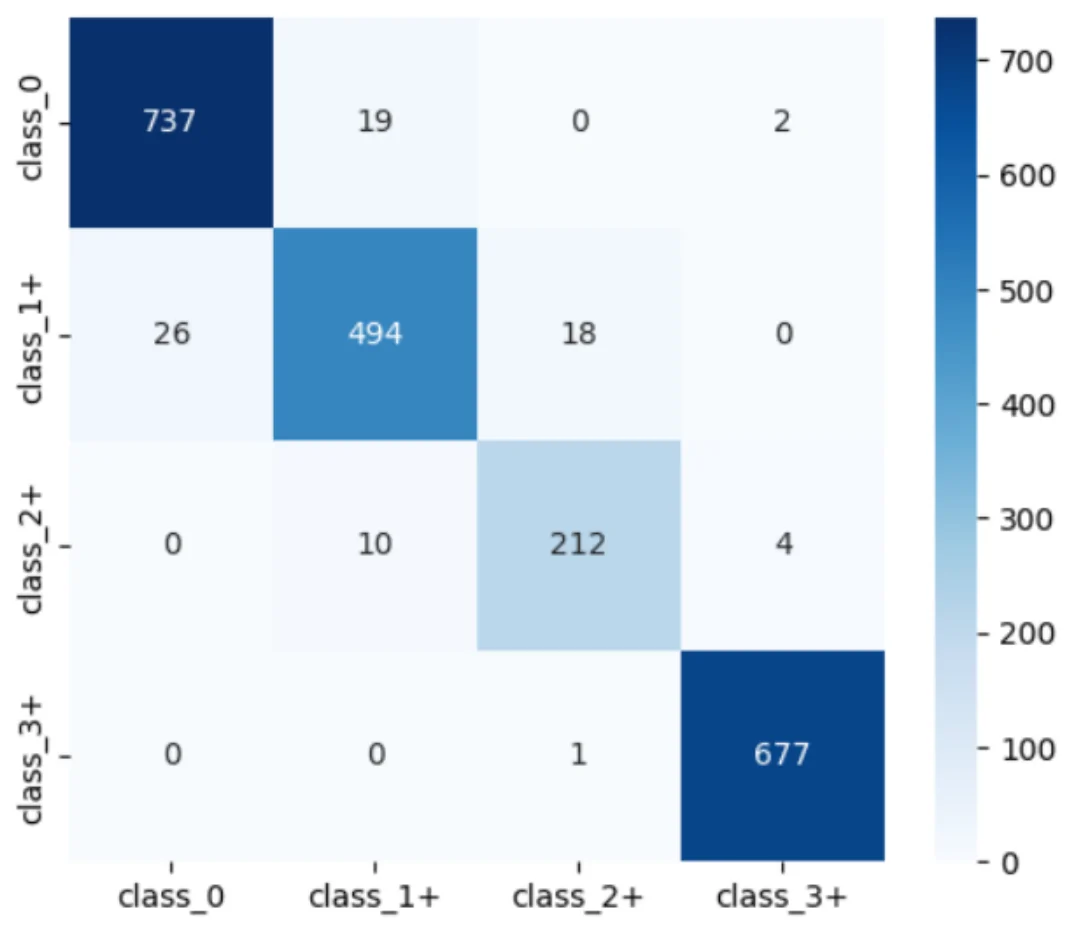
Confusion matrix of the proposed MLFRNet model on the test partition (Seed 42).

Figure 3 shows the confusion matrix obtained during the final evaluation. The matrix provides a detailed view of the correctly and incorrectly classified samples across the four HER2-IHC categories. The model performs particularly well for the two extreme categories, Classes 0 and 3+. The largest bidirectional error count is between Classes 0 and 1+: 19 Class 0 samples were predicted as Class 1+ and 26 Class 1+ samples were predicted as Class 0, giving 45 errors in both directions. Additional confusion is observed between Classes 1+ and 2+, with 18 Class 1+ samples predicted as Class 2+ and 10 Class 2+ samples predicted as Class 1+, giving 28 errors in both directions. For Seed 42, 737 samples from Class 0, 494 from Class 1+, 212 from Class 2+, and 677 from Class 3+ were correctly classified. Table 8 gives the class-wise performance results for seed 42. Sensitivity, specificity, precision, F1-score, AUROC, and PR-AUC are reported for each class. These measures provide a more useful description of class-specific performance than one-vs-rest accuracy, since accuracy in a one-vs-rest setting can be dominated by the large number of true-negative samples.

For class (k), sensitivity, specificity, precision, and F1-score are calculated as

$$Sensitivity_{k}=\frac{TP_{k}}{TP_{k}+FN_{k}},$$

$$Specificity_{k}=\frac{TN_{k}}{TN_{k}+FP_{k}},$$

$$Precision_{k}=\frac{TP_{k}}{TP_{k}+FP_{k}},$$

and

$$F1_{k}=\frac{2\;Precision_{k}\;Sensitivity_{k}}{Precision_{k}+Sensitivity_{k}},$$

where $TP_{k}$, $TN_{k}$, $FP_{k}$, and $FN_{k}$ denote the true-positive, true-negative, false-positive, and false-negative counts for class (k) under the one-vs-rest formulation.

Class 3+ shows the highest class-wise sensitivity and F1-score, whereas the intermediate classes remain more difficult to separate. For Class 0, the sensitivity is 0.9723, and the F1-score is 0.9691. For Class 1+, the corresponding values are 0.9182 and 0.9312, while Class 2+ has a sensitivity of 0.9381 and an F1-score of 0.9278.

For the multiclass ROC analysis, predicted probabilities were used to construct a one-vs-rest ROC curve for each HER2-IHC class. The probability threshold was varied to obtain the corresponding true-positive rate (TPR) and false-positive rate (FPR), defined as

$$TPR=\frac{TP}{TP+FN},\;FPR=\frac{FP}{FP+TN}.$$

The AUROC for class *k* is then obtained from

$$AUROC_{k}=\int_{0}^{1}TPR_{k}\left(FPR\right)\;dFPR.$$

For multiclass ROC (Receiver Operating Characteristic) analysis, the class labels were binarized using a one-vs-rest (OvR) formulation. For each HER2-IHC class, the class-specific AUROC (Area Under the Receiver Operating Characteristic Curve) was calculated using the corresponding binary labels and predicted class probabilities with scikit-learn’s roc_auc_score. The primary multiclass metric was the macro OvR AUROC, defined as the unweighted arithmetic mean of the four class-specific OvR AUROCs. For Seed 42, the macro OvR AUROC was 0.9958 (99.58%).

For precision–recall analysis, average precision (AP) was calculated separately for each HER2-IHC class using scikit-learn’s average_precision_score under the one-vs-rest formulation. Average precision summarizes the precision–recall curve as a weighted sum of precision values at successive thresholds, where the weight assigned to each precision value is the corresponding increase in recall. It can be expressed as

(13) $$\mathrm{AP}=\sum_{n}\left(R_{n}-R_{n-1}\right)P_{n},$$

where $P_{n}$ and $R_{n}$ denote precision and recall at the *n*-th point of the precision–recall curve, respectively, as the decision threshold varies over the predicted scores.

**Figure 4:**
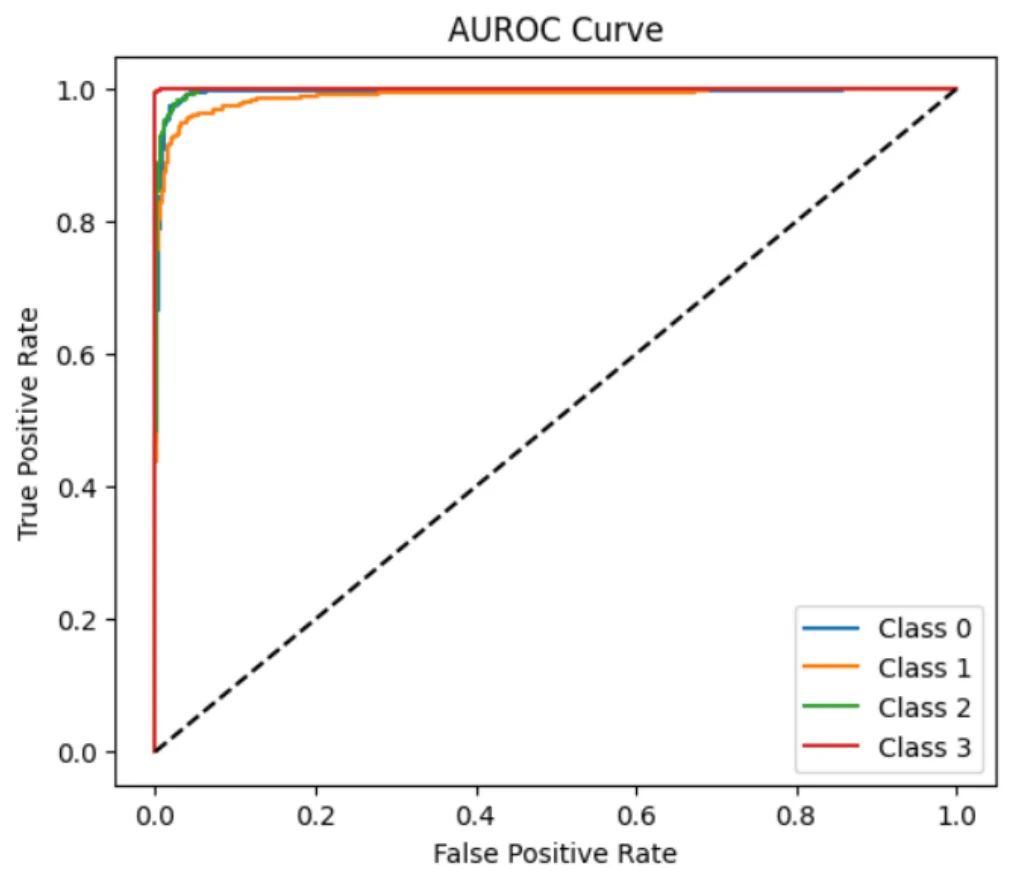
One-versus-rest AUROC curves of the proposed MLFRNet model across the four HER2 classes (Seed 42).

The ROC curves in Figure 4 show high one-vs-rest discrimination across all four HER2 classes. The class-wise AUROC values range from 0.9901 to 1.0000, indicating strong separation of each class from the remaining classes based on the predicted probability outputs.

**Figure 5:**
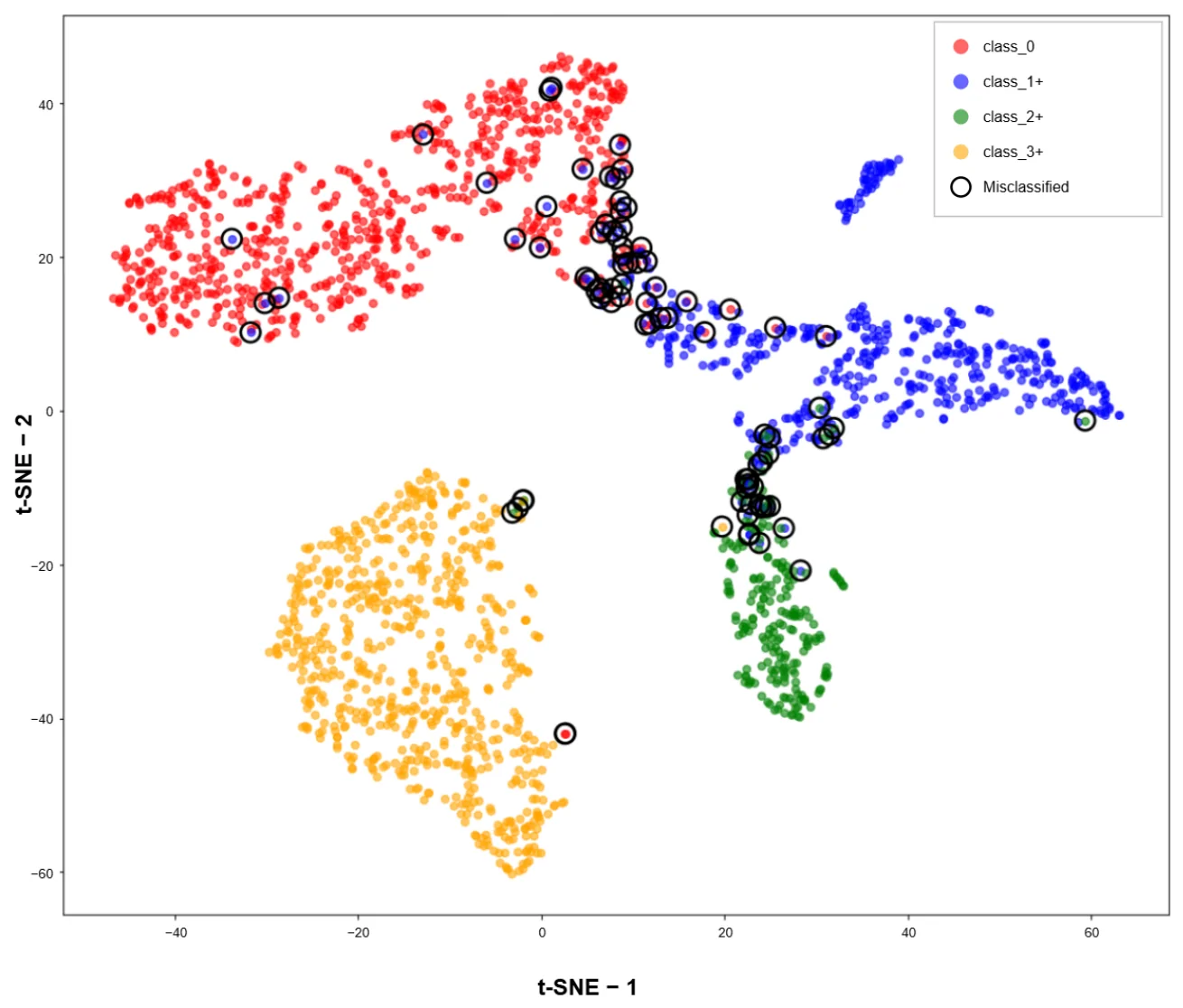
t-SNE visualization of the 2200 test-patch feature representations from MLFRNet (Seed 42). Misclassified samples are highlighted by circles. The visualization is descriptive and is not used as an independent performance measure.

Overall, the class-wise and aggregate discrimination measures indicate strong separation among the four HER2 categories, while the lower performance observed for Classes 1+ and 2+ highlights the remaining difficulty associated with intermediate staining patterns. These findings are also reflected in the feature-space visualization shown in Figure 5. The t-SNE embeddings exhibit relatively distinct distributions for the four classes, with clearer separation for Classes 0 and 3+ and greater overlap between Classes 1+ and 2+. This qualitative observation is consistent with the class-wise diagnostic results and suggests that the intermediate HER2 categories contain more similar visual characteristics.

As a post hoc descriptive check, an extended-training run of 50 epochs was conducted for Seed 42. The model achieved a test accuracy of 96.32% and a macro-OvR AUROC of 99.57%, compared with the selected 15-epoch checkpoint, which achieved 96.36%accuracy and 99.58% AUROC.

### 4.6. Qualitative Analysis

We have considered Grad-CAM and t-SNE visualizations for qualitative analysis. Details of each visualization are as follows.

#### 4.6.1. Grad-CAM Visualization

**Figure 6:**
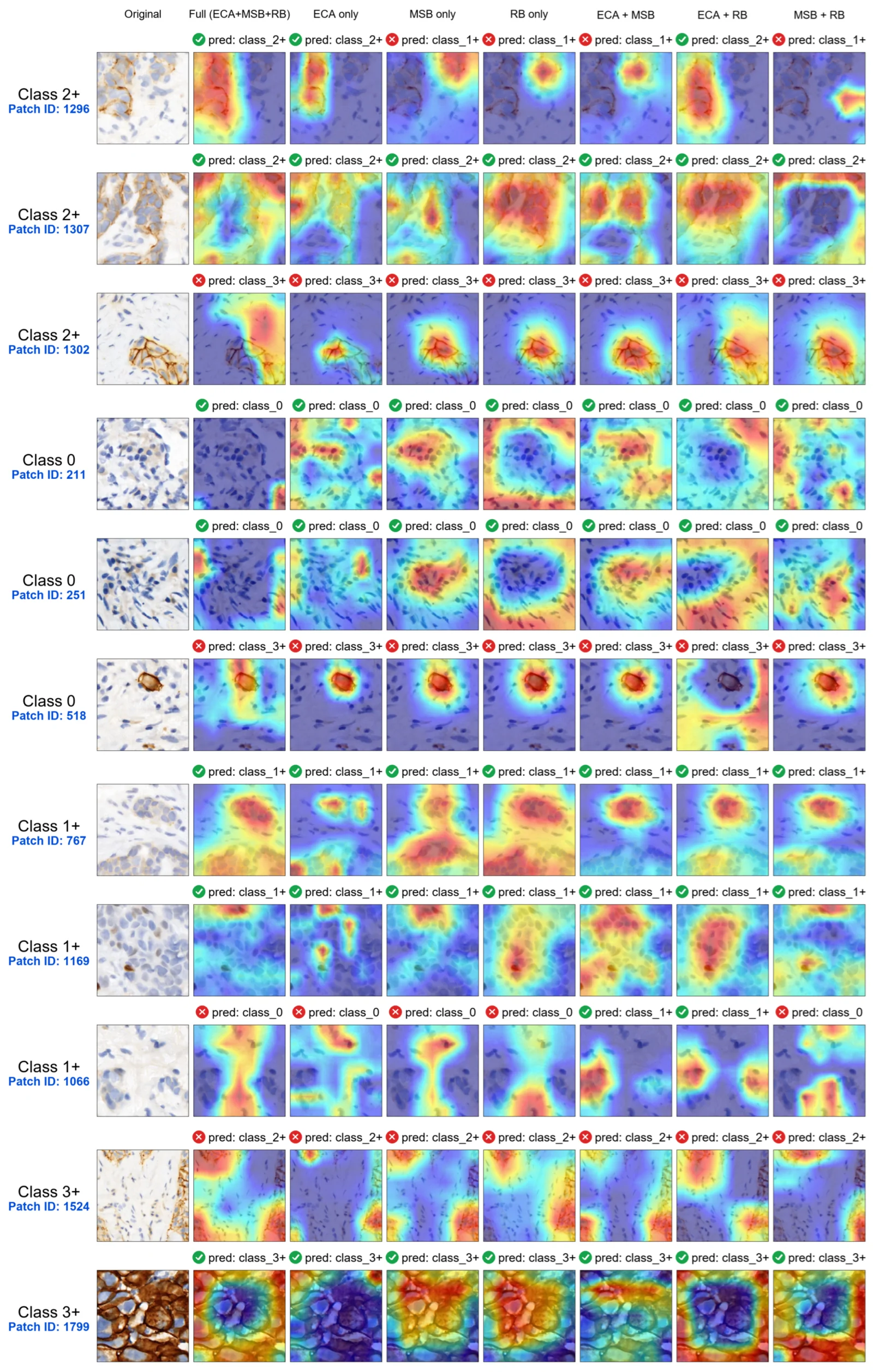
Grad-CAM visualizations of MLFRNet and its ablation variants across the four HER2 classes (Seed 42). The same test patches are shown across all model variants; model-specific predicted classes are indicated for each example. A green check mark indicates that the model predicted class matches the ground-truth label, and a red cross indicates a misclassification.

- **Grad-CAM visualization and example-selection procedure:** Grad-CAM was used to qualitatively examine the spatial attribution patterns of MLFRNet and its ablation variants. The analysis was performed on the official test partition using the saved checkpoints of the seven evaluated configurations: Full (ECA+MSB+RB), ECA only, MSB only, RB only, ECA+MSB, ECA+RB, and MSB+RB. Before Grad-CAM computation, each test patch was resized to 224×224 pixels and normalized using the ImageNet mean (0.485,0.456,0.406) and standard deviation (0.229,0.224,0.225). Grad-CAM was implemented using the pytorch-grad-cam framework. For each configuration, the target layer was selected as the deepest available non-identity feature-refinement module: the Refinement Block (RB) for models containing RB, the Multi-Scale Block (MSB) for models without RB but containing MSB, and ECA for the ECA-only configuration. The final backbone feature layer was used only as a fallback.
- **Example selection:** Examples were selected separately for each HER2 class using the full MLFRNet model (ECA+MSB+RB) as the reference model. For each class, patches were divided into two candidate groups: (i) correctly classified patches, and (ii) misclassified patches whose ground-truth label belonged to that class. Candidate ordering was randomized using a fixed selection seed of 0. Up to three correctly classified and three misclassified patches were selected for each class, with at most 40 candidate patches examined for each group.
- **Cross-variant consistency and quality control:** To ensure that the visual comparison was performed on identical image content, the same physical test patch was evaluated across all seven model variants. A candidate patch was retained only when the Grad-CAM maps generated for all seven variants satisfied the predefined visualization-quality criteria. A map was considered suitable when its normalized activation range was at least 0.35, its spatial standard deviation was at least 0.08, and more than 2% of its pixels had normalized activation values greater than 0.6. Candidates producing a degenerate or near-flat heatmap for any variant were rejected. Consequently, each row in Figure 6 corresponds to the same test patch across all model variants, with the original image shown alongside the corresponding Grad-CAM overlays.
- **Grad-CAM target definition:** For each model variant, the Grad-CAM target was defined as that model’s own predicted class rather than a fixed ground-truth target. The predicted class was determined from the maximum-logit output of the corresponding model and used as the Grad-CAM target. Therefore, when different variants produced different predictions for the same patch, their Grad-CAM maps explain their respective predicted classes rather than a common target class. This distinction is important when comparing attribution patterns across variants because differences may reflect both changes in the learned representation and changes in the prediction being explained. The patch identifiers and model-specific predicted classes are displayed in Figure 6.
- **Interpretation of Grad-CAM maps:** Each Grad-CAM map was independently normalized before visualization. Accordingly, the color intensity represents the relative spatial attribution pattern within an individual map and is not interpreted as a quantitative measure of attribution strength across different images, classes, or model variants. The Grad-CAM results are therefore intended only as qualitative model-attribution visualizations and are not interpreted as quantitative localization measurements or clinically validated localization evidence.

#### 4.6.2. t-SNE Visualization

For the t-SNE analysis in Figure 5, the input features were the 768-dimensional pooled feature vectors after the ConvNeXt-Tiny backbone and the ECA/MSB/RB refinement pathway. All 2200 test patches were used. The analysis used scikit-learn t-SNE with random seed 42, perplexity 30, learning rate set to “auto”, PCA initialization, Euclidean distance, and 1000 optimization iterations. The circled points indicate misclassified test patches. The t-SNE visualization is used only as a descriptive representation-space analysis and is not treated as independent validation of classification performance.

### 4.7. Quantitative Analysis

Table 9 compares the proposed MLFRNet model with internally re-evaluated pre-trained architectures using three prespecified stochastic runs on the official 2200-patch test partition. All baseline models were evaluated using the same experimental protocol as MLFRNet, including the corresponding training/validation partitions, preprocessing and augmentation pipeline, optimizer and learning-rate schedule, batch size, loss function, and validation-based checkpoint-selection procedure. The reported results therefore represent the mean ± standard deviation across three runs rather than a single best-performing seed.

MLFRNet achieved a mean accuracy of 96.10±0.22% and a mean F1-score of 95.30±0.23%. Among the internally re-evaluated baseline models, ConvNeXt-Tiny + Classifier achieved a mean accuracy of 94.24±1.78% and a mean F1-score of 93.12±2.07%. Thus, the difference between MLFRNet and the ConvNeXt-Tiny + Classifier baseline is 1.86 percentage points in mean accuracy and 2.18 percentage points in mean F1-score. Across the other evaluated architectures, the mean accuracy values were within a range beginning at 92.55%.

Across the three prespecified runs, between-run variability differed across model configurations. ConvNeXt-Tiny + Classifier achieved 94.24 ± 1.78% accuracy and 93.12 ± 2.07% macro-F1, whereas MLFRNet achieved 96.10 ± 0.22% accuracy and 95.30 ± 0.23% macro-F1. The observed variability was configuration-dependent, with larger run-to-run variation observed for some partial refinement configurations, including the ECA-only and ECA + MSB variants. Across the three runs, MLFRNet achieved a higher mean accuracy and macro-F1 than the ConvNeXt-Tiny + Classifier baseline, although the magnitude of the difference was not uniform across individual seeds. For example, in Seed 242, the accuracy values were 96.00% for MLFRNet and 96.05% for ConvNeXt-Tiny + Classifier. As only three prespecified runs were evaluated, the reported standard deviations are descriptive of the observed experimental variability.

MLFRNet achieved higher mean accuracy and F1-score than the pretrained architectures re-evaluated under the same experimental protocol. This provides a consistent comparison of the evaluated models. However, generalization to new patients and whole-slide images remains uncertain because patient- and slide-level independence could not be verified from the available dataset, as discussed in the Discussion and Limitations sections.

Table 10 presents a contextual comparison between MLFRNet and previously reported HER2 classification approaches. Nabi et al. [19] reported an accuracy of 94.00% and a macro F1-score of 93.00% using a hybrid ResNet18–EfficientNet-B0 architecture. An explainable deep learning approach based on CNN and DenseNet121 reported an accuracy of approximately 93.00% and an F1-score of 92.00% [13]. Hasan et al. [30] reported an accuracy of 96.15% and an F1-score of 94.82% for GEM-Net(Tiny). In comparison, MLFRNet achieved a mean accuracy of 96.10±0.22% and a mean F1-score of 95.30±0.23% across three prespecified stochastic runs.

As these previously reported studies may differ in dataset partitioning, preprocessing procedures, training strategies, model-selection procedures, and evaluation protocols, their results are presented only for contextual comparison and are not treated as direct head-to-head comparisons. The models evaluated in Table 9 serve as baselines for comparison with MLFRNet because they were evaluated using the same experimental protocol.

### 4.8. Input-Resolution Sensitivity

The original HER2-IHC patches have a native resolution of 1024×1024 pixels. To examine the sensitivity of MLFRNet to the input resolution, additional experiments were conducted using the same Seed-42 training/validation partition and the same 2200-patch held-out test partition at three input resolutions: 224×224, 384×384, and 512×512. The 224×224 setting corresponds to the primary Seed-42 configuration and achieved 96.36% test accuracy and 95.57% macro-F1. The 384×384 setting achieved 96.14% accuracy and 95.44% macro-F1, while the 512×512 setting achieved 96.05% accuracy and 95.22% macro-F1.The results are summarized in Table 11.

All three resolution settings used the same maximum 15-epoch training schedule, loss formulation, optimizer, learning-rate schedule, and validation-based checkpoint-selection procedure. Checkpoint selection was performed using validation accuracy only, without using the official test partition for model selection. The 384×384 experiment used a batch size of 32, while the 512×512 experiment used a batch size of 16 to accommodate the increased memory requirements of the higher-resolution inputs.

Given the cumulative spatial stride of 32 in the ConvNeXt-Tiny backbone, the resulting feature maps had dimensions of 7×7×768, 12×12×768, and 16×16×768 for input resolutions of 224×224, 384×384, and 512×512, respectively. These resolution experiments were conducted as a single-seed (Seed 42) sensitivity analysis; therefore, the results are interpreted as exploratory comparisons across input resolutions rather than as estimates of between-seed variability.

The results show only small differences across the evaluated input resolutions. Relative to the primary 224×224 setting, the 384×384 configuration produced a 0.22 percentage-point lower accuracy and a 0.13 percentage-point lower macro-F1, while the 512×512 configuration produced a 0.31 percentage-point lower accuracy and a 0.35 percentage-point lower macro-F1. Thus, increasing the input resolution did not produce a measurable performance improvement in this Seed-42 sensitivity analysis. The 224×224 configuration was retained as the primary setting as it provided comparable classification performance while requiring lower computational resources.

## 5. Discussion and Limitations

From a computational perspective, MLFRNet contains 41.447 million trainable parameters and requires approximately 18.465 G MACs for a 224×224 input. The measured inference latency, throughput, model-file size, and peak GPU memory provide a quantitative characterization of the computational requirements of the proposed architecture. These measurements should not be interpreted as demonstrating computational efficiency relative to competing architectures because controlled complexity and runtime comparisons under identical hardware and software conditions were not performed. In particular, the Refinement Block contains two conventional 3×3 convolutions operating on 768 channels and therefore contributes to the overall computational cost. Future work should investigate the accuracy–complexity trade-off through controlled comparisons with lightweight or compressed architectures. The interpretation of the multi-scale module is intentionally limited to spatial context. The 3×3, 5×5, and 7×7 branches operate on the same final 7×7×768 feature representation and therefore are not interpreted as direct cellular, cell-cluster, or tissue-level representations. Establishing such biological correspondence would require additional evidence, such as receptive-field analysis, multi-stage feature experiments, or quantitative localization using expert-annotated regions. Accordingly, this study describes these branches in terms of their relative spatial neighborhood extents rather than assigning predefined biological scales. The Grad-CAM analysis was not quantitatively validated against independent pathologist-annotated membrane-region masks. Therefore, the highlighted regions are interpreted as model-attribution maps rather than clinically validated diagnostic localizations. Quantitative evaluation against expert-annotated membrane regions remains an important direction for future work. The study has limitations related to dataset generalization and clinical interpretation. The evaluation is performed at the patch level and therefore does not establish whole-slide or patient-level diagnostic performance. An independent external cohort was not available for validation, limiting assessment of generalization across institutions and patient populations. Patient- and whole-slide-level independence of the current partitions also cannot be verified from the public release because patient/WSI identifiers and source coordinates are unavailable. We therefore do not interpret the reported accuracy as an unbiased estimate of patient-level generalization. The image-content audit found no exact byte-level duplicates or perceptual–hash candidate pairs between the development and test partitions, but this audit cannot establish patient/WSI independence. Future work should evaluate MLFRNet using verifiable patient- or whole-slide-disjoint datasets and investigate slide-level aggregation for clinically meaningful HER2 IHC scoring.

## 6. Conclusions

In this study, we presented the Multi-scale Local Feature Refinement Network (MLFRNet), which combines ECA, MSB, and RB blocks for HER2 score classification. The architecture uses ECA for channel-wise feature recalibration, multi-scale depthwise convolutions for complementary spatial context, and a residual refinement block for further feature refinement. On the HER2-IHC-40× benchmark, MLFRNet achieved a mean accuracy of 96.10±0.22% and a mean macro F1-score of 95.30±0.23% across three prespecified stochastic runs. Additional single-seed sensitivity experiments at 384×384 and 512×512 produced accuracies of 96.14% and 96.05%, respectively, with similar macro-F1 values to the primary 224×224 setting. Internally re-evaluated pretrained models were compared under the same experimental protocol, whereas previously published methods were treated only as contextual comparisons because their experimental settings may differ. The study is limited by the patch-level nature of the benchmark and the inability to independently verify patient- or whole-slide-level partition independence from the public release. Future work should therefore evaluate MLFRNet on verifiable patient- or whole-slide-disjoint datasets and assess slide-level aggregation for clinically meaningful HER2 IHC scoring.

## 7. Code Availability

The MLFRNet source code and associated experimental resources will be made publicly available in the official GitHub repository: https://github.com/mohan-sai-77/MLFRNet (accessed on 14 October 2026) upon publication of this paper. The planned release will include the training and evaluation implementation, experimental configuration files, random-seed and data-partition specifications, validation-based checkpoint-selection procedures, metric-calculation scripts, prediction outputs, computational profiling scripts, and software environment information required to reproduce the reported experiments. The release will also include the scripts and supporting results for the image-content similarity audit, including perceptual-hash (pHash/dHash) screening, structural similarity index (SSIM) analysis, ConvNeXt-Tiny embedding-based similarity analysis, and documentation of the ten image pairs identified with cosine similarity greater than 0.98. The repository will document the checkpoints and configurations associated with the primary 224 × 224 experiments and the 384 × 384 and 512 × 512 resolution-sensitivity experiments, including the corresponding random seeds, training epochs, validation performance, and checkpoint-selection criteria. 

## Figures and Tables

**Table 1:** Feature evolution and functional role of the proposed MLFRNet architecture.

Stage	Operation	Input Size	hlOutput Size	Learned Representation	Representation Level	Functional Role
Input	RGB Image	3 × 224 × 224	3 × 224 × 224	Raw image representation	Pixel Level	Provides the normalized model input.
ConvNeXt Stage 1	Stem + CN Blocks	3 × 224 × 224	96 × 56 × 56	Early feature representation	Early Feature Level	Extracts initial spatial features.
ConvNeXt Stage 2	Downsampling + CN Blocks	96 × 56 × 56	192 × 28 × 28	Local feature representation	Local Feature Level	Learns structured local features.
ConvNeXt Stage 3	Downsampling + CN Blocks	192 × 28 × 28	384 × 14 × 14	Intermediate feature representation	Intermediate Feature Level	Builds deeper spatial context.
ConvNeXt Stage 4	Downsampling + CN Blocks	384 × 14 × 14	768 × 7 × 7	High-level feature representation	High-level Feature Level	Produces the final backbone features.
ECA	Efficient Channel Attention	768 × 7 × 7	768 × 7 × 7	Channel-wise weighted representation	Channel Attention Level	Reweights channel responses.
MSB (3×3)	Depthwise Convolution	768 × 7 × 7	768 × 7 × 7	Small-neighborhood spatial features	Small Spatial Neighborhood	Captures relatively local context.
MSB (5×5)	Depthwise Convolution	768 × 7 × 7	768 × 7 × 7	Intermediate-neighborhood spatial features	Intermediate Spatial Neighborhood	Captures broader spatial context.
MSB (7×7)	Depthwise Convolution	768 × 7 × 7	768 × 7 × 7	Large-neighborhood spatial features	Large Spatial Neighborhood	Captures the broadest MSB context.
Concatenation	Multi-scale Fusion	3 × (768 × 7 × 7)	2304 × 7 × 7	Multi-scale feature representation	Multi-scale Feature Level	Combines the three branch outputs.
1×1 Conv	Channel Projection	2304 × 7 × 7	768 × 7 × 7	Projected multi-scale representation	Feature Projection Level	Restores the original channel dimension.
RB	Refinement Block	768 × 7 × 7	768 × 7 × 7	Refined feature representation	Feature Refinement Level	Refines features through residual convolutions.
GAP	Global Average Pooling	768 × 7 × 7	768	Pooled feature representation	Patch summary level	Aggregates spatial features into one vector.
FC Head	Classification Head	768	4	Class prediction representation	Decision Level	Predicts four HER2 score classes.

**Table 2:** Class-wise distribution of HER2-IHC image patches in the predefined training pool and official test partition of the HER2-IHC-40× dataset.

HER2 Class	Training Samples	Test Samples
0	3031	758
1+	2151	538
2+	905	226
3+	2710	678
Total	8797	2200

**Table 4:** Architectural ablation results across three runs (seeds 42, 142, and 242), reported as mean ± sample standard deviation on the official 2200-patch test set. The same training/validation split was used for all configurations within each seed.

Base Model	Combination Blocks	Accuracy (%)	F1-Score (%)
ConvNeXt-Tiny	–	94.24 ± 1.78	93.12 ± 2.07
ConvNeXt-Tiny	ECA	94.76 ± 2.11	93.72 ± 2.57
ConvNeXt-Tiny	MSB	95.68 ± 0.14	94.70 ± 0.11
ConvNeXt-Tiny	RB	95.53 ± 0.30	94.58 ± 0.43
ConvNeXt-Tiny	ECA + MSB	94.69 ± 1.78	93.84 ± 2.02
ConvNeXt-Tiny	ECA + RB	95.68 ± 0.14	94.66 ± 0.18
ConvNeXt-Tiny	MSB + RB	95.92 ± 0.39	95.08 ± 0.51
ConvNeXt-Tiny	ECA + MSB (3,5) + RB	95.82 ± 0.23	94.95 ± 0.42
ConvNeXt-Tiny	ECA + MSB (3,7) + RB	95.98 ± 0.18	95.01 ± 0.29
ConvNeXt-Tiny	ECA + MSB (5,7) + RB	95.80 ± 0.19	94.88 ± 0.18
ConvNeXt-Tiny	ECA + MSB + RB	96.10 ± 0.22	95.30 ± 0.23

**Table 5:** Parameter count of the MLFRNet components.

Component	Parameter Count
ConvNeXt-Tiny Backbone	28,589,128
ECA	5
MSB	1,839,360
RB	10,619,904
Classifier	398,340

**Table 6:** Seed-wise classification performance of MLFRNet across three prespecified stochastic runs on the 2200-patch official test partition.

Seed	Accuracy (%)	Precision (%)	Recall (%)	F1-Score (%)	AUROC (%)	Kappa
42	96.36	95.49	95.68	95.57	99.58	0.9492
142	95.95	95.14	95.14	95.14	99.51	0.9435
242	96.00	95.38	95.04	95.20	99.61	0.9441
**Mean ± SD**	**96.10 ± 0.22**	**95.34 ± 0.18**	**95.29 ± 0.34**	**95.30 ± 0.23**	**99.57 ± 0.05**	**0.9456 ± 0.0031**

**Table 7:** Computational characteristics of the proposed MLFRNet model.

Characteristic	Measured Value
Trainable parameters	41.447 M
Non-trainable parameters	0
MACs	18.465 G
FLOPs	36.930 G
Model file size	158.28 MB
Peak GPU memory (allocated)	920.4 MB
Peak GPU memory (reserved)	1145.0 MB
Latency, batch size 1	12.421 ± 4.258 ms
Throughput, batch size 1	80.5 images/s
Latency, batch size 32	134.128 ± 1.509 ms
Throughput, batch size 32	238.6 images/s
Hardware	NVIDIA Tesla T4

**Table 8:** Class-wise classification performance of MLFRNet on the predefined official test partition for Seed 42.

Class	Sensitivity	Specificity	Precision	F1-score	AUROC	PR-AUC
Class 0	0.9723	0.9820	0.9659	0.9691	0.9956	0.9921
Class 1+	0.9182	0.9826	0.9446	0.9312	0.9901	0.9769
Class 2+	0.9381	0.9904	0.9177	0.9278	0.9975	0.9806
Class 3+	0.9985	0.9961	0.9912	0.9949	1.0000	1.0000
**Macro Avg.**	0.9568	0.9877	0.9549	0.9557	0.9958	0.9874

**Table 9:** Comparison of the proposed MLFRNet model with internally re-evaluated pre-trained models using three prespecified stochastic runs on the official 2200-patch test partition. Values are reported as mean ± standard deviation.

Model	Accuracy (%)	F1-Score (%)
ResNet18 [9]	92.59±0.32	92.01±1.06
ResNet50 [9]	93.67±0.32	92.86±0.10
EfficientNet-B0 [12]	93.47±0.52	92.87±0.34
MobileNetV2 [25]	93.66±0.29	92.88±0.75
MobileNetV3-L [26]	93.48±0.32	93.15±0.16
DenseNet169 [10]	93.51±0.33	92.81±0.96
EfficientViT [27]	92.55±0.28	91.46±0.32
DeiT-Tiny [28]	93.79±0.37	92.75±0.36
SwiftFormer-XS [29]	93.50±0.50	92.84±0.29
ConvNeXt-Tiny + Classifier [11]	94.24±1.78	93.12±2.07
**Proposed MLFRNet**	96.10±0.22	95.30±0.23

**Table 10:** Contextual comparison of the proposed method with existing HER2 classification approaches.

Reference	Accuracy (%)	F1-Score (%)	Model
Nabi et al. [13]	93.00	92.00	DenseNet121 + CNN
Nabi et al. [19]	94.00	93.00	ResNet18 + EfficientNet-B0
Hasan et al. [30]	96.15	94.82	GEM-Net(Tiny)
Proposed Model	96.10 ± 0.22	95.30 ± 0.23	ConvNeXt-Tiny+MLFR

**Table 11:** Input-resolution sensitivity analysis of MLFRNet on the same official 2200-patch test partition using the Seed-42 training/validation partition.

Input Resolution	Test Accuracy (%)	Macro-F1 (%)
224 × 224	96.36	95.57
384 × 384	96.14	95.44
512 × 512	96.05	95.22

## Data Availability

The HER2-IHC-40× histopathological dataset analyzed during the current study is available in the Zenodo repository at: https://zenodo.org/records/15179608 (accessed on 14 October 2026).

## Supplementary Materials

The following supporting information can be downloaded at: https://www.mdpi.com/article/10.3390/diagnostics16213614/s1, The material contains of Table S1: Seed-wise accuracy and macro-F1 values underlying the reported three-run summaries (S1-A: loss-function ablation; S1-B: architectural ablation; S1-C: evaluated baseline models); Table S2: Image-content overlap audit of the ten development–test image pairs with ConvNeXt-Tiny embedding cosine similarity above 0.98; Figure S1: Contact sheet of the ten development–test pairs with cosine similarity above 0.98.
