# Supplementary material for: MLFRNet: Multi-Scale Local Feature Refinement Network Using Multi-Scale Depthwise Convolution for HER2 Score Classification
Source: Diagnostics (Basel). 2026 Nov 1;16(21):3614. doi: 10.3390/diagnostics16213614 (PMC13761575; doi:10.3390/diagnostics16213614)
Supplement: Supplementary file 1 [file diagnostics-16-03614-s001.zip › diagnostics-4487418-supplementary.pdf]

Supplementary Materials : MLFRNet : Multi-scale Local Feature Refinement Network using Multi-scale Depthwise Convolution for HER2 score classification

Mohan Venkata Sai Valluru [1], Bala Venkateswarlu Isunuri [2,*] and Sibendu Samanta [3]

Mohan Venkata Sai Valluru, Bala Venkateswarlu Isunuri and Sibendu Samanta

Supplementary Table S1. Seed-wise results underlying the reported three-run summaries

The individual seed-wise evaluation records below provide the directly observed values used to verify the three-run summaries reported in the main manuscript. All three-run mean ± SD summaries use the sample standard deviation.

S1-A. Loss-function ablation

Configuration	Seed	Accuracy (%)	Macro-F1 (%)
ConvNeXt-Tiny + CE loss	42	93.14	91.67
ConvNeXt-Tiny + CE loss	142	93.64	92.35
ConvNeXt-Tiny + CE loss	242	92.50	91.43
ConvNeXt-Tiny + Focal loss	42	93.14	91.70
ConvNeXt-Tiny + Focal loss	142	92.64	91.28
ConvNeXt-Tiny + Focal loss	242	92.86	91.80
ConvNeXt-Tiny + CE + Label Smoothing (CELS)	42	93.18	91.65
ConvNeXt-Tiny + CE + Label Smoothing (CELS)	142	93.50	92.44
ConvNeXt-Tiny + CE + Label Smoothing (CELS)	242	93.45	92.60
ConvNeXt + Classifier + CE + Label Smoothing + Focal	42	94.18	93.03
ConvNeXt + Classifier + CE + Label Smoothing + Focal	142	92.50	91.10
ConvNeXt + Classifier + CE + Label Smoothing + Focal	242	96.05	95.24

The corresponding three-run summaries are 93.09 ± 0.57% accuracy and 91.82 ± 0.48% macro-F1 for CE; 92.88 ± 0.25% and 91.59 ± 0.28% for focal loss; 93.38 ± 0.17% and 92.23 ± 0.51% for CELS; and 94.24 ± 1.78% and 93.12 ± 2.07% for the composite loss baseline.

S1-B. Architectural ablation

Configuration	Seed	Accuracy (%)	Macro-F1 (%)
ConvNeXt-Tiny, baseline	42	94.18	93.03
ConvNeXt-Tiny, baseline	142	92.50	91.10
ConvNeXt-Tiny, baseline	242	96.05	95.24
ConvNeXt-Tiny + ECA	42	95.91	95.16
ConvNeXt-Tiny + ECA	142	92.32	90.75
ConvNeXt-Tiny + ECA	242	96.05	95.25
ConvNeXt-Tiny + MSB	42	95.55	94.57
ConvNeXt-Tiny + MSB	142	95.82	94.78
ConvNeXt-Tiny + MSB	242	95.68	94.74
ConvNeXt-Tiny + RB	42	95.86	95.00
ConvNeXt-Tiny + RB	142	95.27	94.14
ConvNeXt-Tiny + RB	242	95.45	94.61
ConvNeXt-Tiny + ECA + MSB	42	95.55	94.54
ConvNeXt-Tiny + ECA + MSB	142	92.64	91.56
ConvNeXt-Tiny + ECA + MSB	242	95.87	95.42
ConvNeXt-Tiny + ECA + RB	42	95.55	94.54
ConvNeXt-Tiny + ECA + RB	142	95.68	94.57
ConvNeXt-Tiny + ECA + RB	242	95.82	94.86
ConvNeXt-Tiny + MSB + RB	42	96.27	95.50
ConvNeXt-Tiny + MSB + RB	142	95.50	94.51
ConvNeXt-Tiny + MSB + RB	242	96.00	95.23
ConvNeXt-Tiny + MSB (3,5) + Classifier	42	95.59	94.52
ConvNeXt-Tiny + MSB (3,5) + Classifier	142	95.82	94.98
ConvNeXt-Tiny + MSB (3,5) + Classifier	242	96.05	95.35

Continued on next page

Configuration	Seed	Accuracy (%)	Macro-F1 (%)
ConvNeXt-Tiny + MSB (3,7) + Classifier	42	95.82	94.72
ConvNeXt-Tiny + MSB (3,7) + Classifier	142	95.95	95.03
ConvNeXt-Tiny + MSB (3,7) + Classifier	242	96.18	95.29
ConvNeXt-Tiny + MSB (5,7) + Classifier	42	95.95	95.00
ConvNeXt-Tiny + MSB (5,7) + Classifier	142	95.59	94.68
ConvNeXt-Tiny + MSB (5,7) + Classifier	242	95.86	94.97
ConvNeXt-Tiny + ECA + MSB + RB (MLFRNet)	42	96.36	95.57
ConvNeXt-Tiny + ECA + MSB + RB (MLFRNet)	142	95.95	95.14
ConvNeXt-Tiny + ECA + MSB + RB (MLFRNet)	242	96.00	95.20

Note: The architecture labels above follow the configuration terminology used in the main manuscript tables. The same seed-wise records are used for the corresponding mean ± SD summaries.

S1-C. evaluated baseline models

Configuration	Seed	Accuracy (%)	Macro-F1 (%)
ResNet18	42	92.50	90.80
ResNet18	142	92.95	92.77
ResNet18	242	92.32	92.47
ResNet50	42	93.95	92.92
ResNet50	142	93.73	92.74
ResNet50	242	93.32	92.92
EfficientNet-B0	42	93.55	92.72
EfficientNet-B0	142	93.95	93.26
EfficientNet-B0	242	92.91	92.62
MobileNetV2	42	93.64	92.95
MobileNetV2	142	93.96	93.59
MobileNetV2	242	93.39	92.10
MobileNetV3-L	42	93.82	92.97
MobileNetV3-L	142	93.18	93.29
MobileNetV3-L	242	93.45	93.19
DenseNet169	42	93.77	92.51
DenseNet169	142	93.14	92.04
DenseNet169	242	93.63	93.88
EfficientViT	42	92.55	91.43
EfficientViT	142	92.27	91.15
EfficientViT	242	92.82	91.79
DeiT-Tiny	42	94.01	93.00
DeiT-Tiny	142	94.00	92.90
DeiT-Tiny	242	93.36	92.34
SwiftFormer-XS	42	93.81	92.94
SwiftFormer-XS	142	92.92	92.51
SwiftFormer-XS	242	93.77	93.06
ConvNeXt + Classifier	42	94.18	93.03
ConvNeXt + Classifier	142	92.50	91.10
ConvNeXt + Classifier	242	96.05	95.24

Supplementary Table S2. Image-content overlap audit of the ten cosine-similarity flags

The cross-partition audit compared 8,797 development patches against the 2,200 official test patches. SHA-256 exact matching found zero exact byte-level duplicates. The pHash/dHash nearest-neighbor screening used a predefined Hamming-distance threshold of $\leq 10/64$ bits, and the screening produced no candidate pairs within the threshold. SSIM was evaluated after deterministic grayscale conversion and resizing to 256×256 pixels, with SSIM ≥ 0.90 used as the flag threshold. A complementary frozen ImageNet-pretrained ConvNeXt-Tiny embedding analysis used 224×224 inputs, 768-dimensional pooled features, L2 normalization, nearest-neighbor matching, and a cosine-similarity flag threshold of > 0.98.

Ten pairs exceeded the cosine-similarity flag threshold. All ten were visually reviewed. The flagged patches showed similar broad histopathological morphology but different cellular/spatial arrangements and low SSIM, and no obvious duplicated content was observed. They were therefore retained as similarity flags rather than classified as duplicates. The audit evaluates image-content similarity only and does not establish patient- or whole-slide-level independence.

Pair	Cosine	SSIM	pHash	dHash	Class pair	Visual assessment	Status
1	0.984602	0.323435	36	32	1+ ↔ 1+	Visually distinct; similar broad morphology but different cellular/spatial arrangement.	Similarity flag; retained, not classified as duplicate.
2	0.984059	0.241646	26	26	0 ↔ 0	Visually distinct; no duplicate content observed.	Similarity flag; retained, not classified as duplicate.
3	0.983972	0.325517	34	34	1+ ↔ 1+	Visually distinct; similar broad morphology but different cellular/spatial arrangement.	Similarity flag; retained, not classified as duplicate.
4	0.983011	0.271423	34	33	0 ↔ 0	Visually distinct; no duplicate content observed.	Similarity flag; retained, not classified as duplicate.
5	0.982051	0.360982	30	40	1+ ↔ 1+	Visually distinct; similar broad morphology but different cellular/spatial arrangement.	Similarity flag; retained, not classified as duplicate.
6	0.981200	0.280625	38	40	0 ↔ 1+	Visually distinct; different class pair and low pixel-level similarity.	Similarity flag; retained, not classified as duplicate.
7	0.980855	0.340643	34	31	1+ ↔ 1+	Visually distinct; similar broad morphology but different cellular/spatial arrangement.	Similarity flag; retained, not classified as duplicate.
8	0.980802	0.335790	28	32	1+ ↔ 1+	Visually distinct; similar broad morphology but different cellular/spatial arrangement.	Similarity flag; retained, not classified as duplicate.
9	0.980500	0.255766	30	27	1+ ↔ 1+	Visually distinct; no duplicate content observed.	Similarity flag; retained, not classified as duplicate.
10	0.980277	0.230695	30	28	0 ↔ 0	Visually distinct; no duplicate content observed.	Similarity flag; retained, not classified as duplicate.

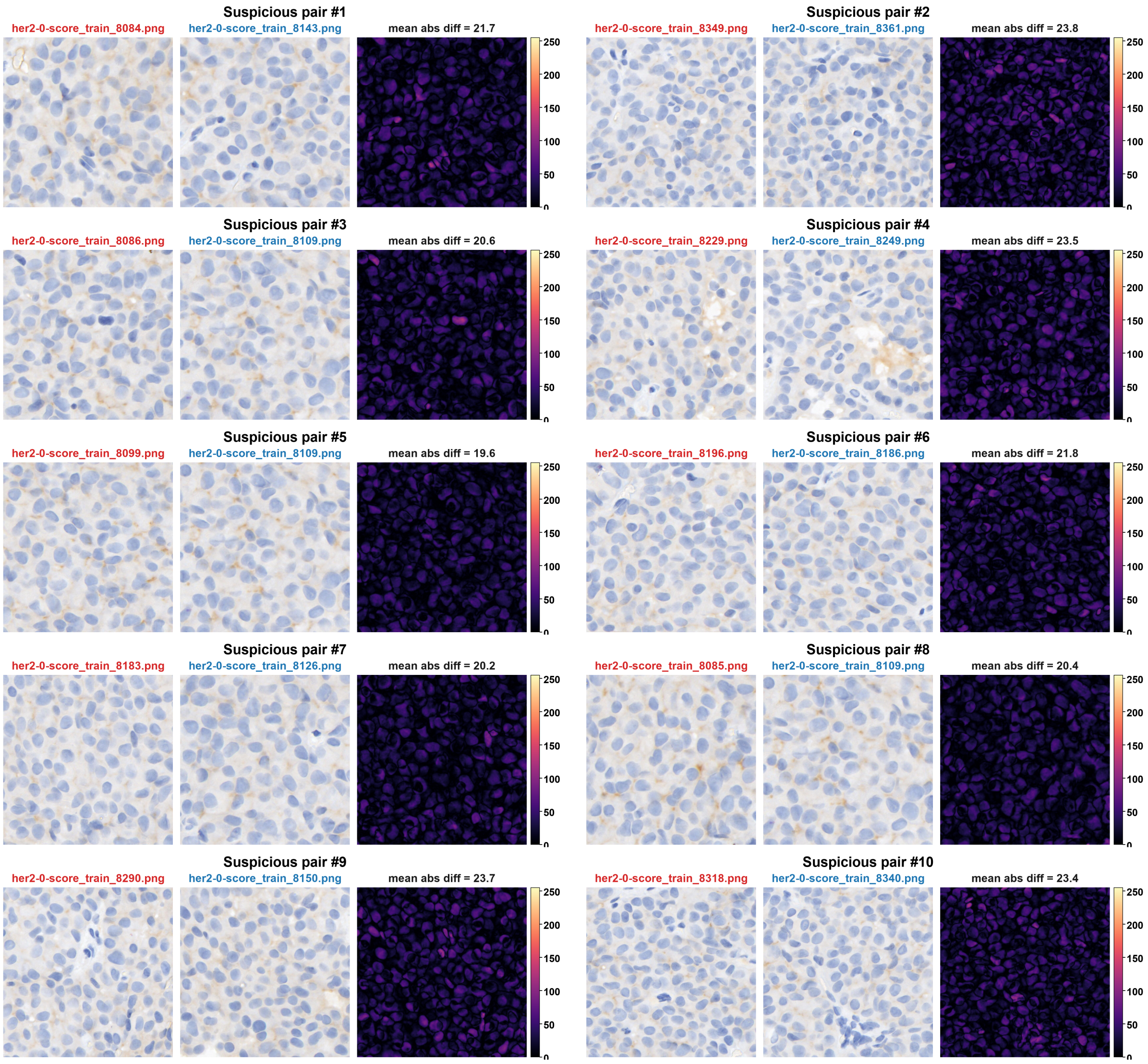

Figure S1: Contact sheet of the ten development–test pairs with cosine similarity > 0.98. Each pair was visually reviewed. The displayed cosine similarity, SSIM, pHash, and dHash values are the audit measurements; the flagged pairs were retained as similarity flags rather than classified as duplicates.

Supplementary audit note

The embedding audit produced a maximum nearest-neighbor cosine similarity of 0.9846019745, with mean 0.8853962784, median 0.8921999037, 95th percentile 0.9566204429, and 99th percentile 0.9715013808. The number of flagged pairs was 977 for cosine similarity > 0.90, 187 for > 0.95, 10 for > 0.98, and zero for > 0.99, > 0.995, and > 0.999. Of the ten pairs above 0.98, nine were same-class pairs and one was a different-class pair. These descriptive audit statistics support the reporting in the main manuscript but do not establish patient- or whole-slide-level independence.
